# Peralkynylated Tetraazaacene Derivatives

**DOI:** 10.1002/chem.201904087

**Published:** 2019-12-16

**Authors:** Hilmar Reiss, Frank Rominger, Jan Freudenberg, Uwe H. F. Bunz

**Affiliations:** ^1^ Organisch-Chemisches Institut Ruprecht-Karls-Universität Heidelberg Im Neuenheimer Feld 270 69120 Heidelberg Germany; ^2^ Centre for Advanced Materials Im Neuenheimer Feld 225 69120 Heidelberg Germany; ^3^ InnovationLab Speyerer Strasse 4 69115 Heidelberg Germany

**Keywords:** *N*-heteropentacenes, peralkynylation, *peri* interactions, stabilization, X-ray diffraction

## Abstract

The synthesis of a decaethynylated tetraazapentacene is described. It was obtained by a combination of condensation reactions giving the two pyrazine rings and subsequent consecutive Stille‐type couplings. This is the first example of any higher (hetero)acene that is peralkynylated. The presence of the four nitrogen atoms removes the *peri* interaction of the substituted alkyne groups, giving this rock‐stable and highly twisted heteroacene.

## Graphical abstract

**Rock‐stable and highly twisted heteroacene**: The synthesis of a decaethynylated tetraazapentacene is described. It was obtained by a combination of condensation reactions giving the two pyrazine rings and subsequent consecutive Stille‐type couplings. This is the first example of any higher (hetero)acene that is peralkynylated (see scheme).

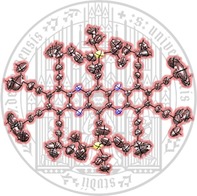

Herein, we disclose the synthesis of an octaalkynylated tetraazatetracene and a decaalkynylated tetraazapentacene. Peralkynylated representatives are known of some aromatic systems; the first ever described was Vollhardt's hexaethynylbenzene, prepared from hexabromobenzene and TMS acetylene in a sixfold Sonogashira reaction.^1^ Later, tetraethynylethylenes were prepared by Hopf and co‐workers^2^ and by Diederich and co‐workers,^3^ followed by tetraalkynylated cyclobutadiene complexes achieved by Bunz and co‐workers^4^ as well as peralkynylated cyclopentadienyl species either as radical (Rubin)^5^ or stabilized as cymantrene or ferrocene.^6^

However, for larger aromatic systems, such as naphthalene or anthracene, peralkynylated species are unknown—attempts of alkynylation lead to annulation reactions due to severe *peri* interactions of the alkynes—yet a peralkenylated naphthalene has been prepared by de Meijere and co‐workers.^7^ To solve this issue, the offending *peri* interactions will have to be removed by substitution of a C‐CC‐R‐group through a nitrogen. As a consequence, peralkynylated phenazine and quinoxaline are known.^8^ However, none of the larger peralkynylated *N*‐heteroacenes have ever been prepared. Herein, we disclose the synthesis of the higher homologues, a peralkynylated tetraazatetracene and tetraazapentacene derivative.

Starting from diamine **1**,^9^ reaction with tetrabromoorthoquinone under acidic conditions furnished the thiadiazole **2**, which, upon Stille reaction with the stannylated acetylenes followed by MnO_2_ oxidation of the intermediate *N*,*N′*‐dihydro‐compound, gave the hexaethynylated phenazinthiadiazoles **3 a** and **b** (Scheme 1). Attempts to open the thiadiazole ring with LiAlH_4_ resulted in low yields, with zinc in acetic acid lead to decomposition. However, SmI_2_ smoothly opened the thiadiazole ring to give the diamines **4 a** or **b** quantitatively. Compound **4 a** was easily coupled with dione **5**, resulting in the first octaalkynylated tetraazatetracene **6** as a turquoise solid. The subsequent synthesis of the symmetrical tetraazapentacene motif was more challenging. The coupling of **4 b** with a second mole of tetrabromoorthoquinone gave the compound **7 b‐H_2_**—after reduction with tin chloride—in only 8 % yield, whereas the reaction with **4 a** gave the product **7 a** (see the Supporting Information) in 5 %, probably due to the lower stability of the TMS groups. The (high yielding) reduction is critical to allow the next fourfold Stille reaction, because the oxidized form of **7 b‐H_2_** oxidizes the Pd catalyst. The formed *N*,*N′*‐dihydrotetraazacene **8‐H_2_** is oxidized easily into the target compound **8**. With this sequence, the first peralkynylated azapentacene has been prepared as a red crystalline material.

**Scheme 1:**
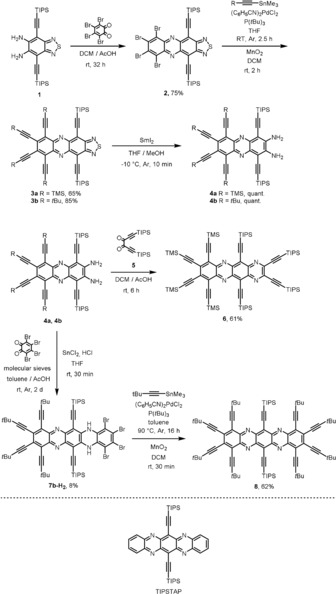
Synthesis of the peralkynylated tetraazaacenes **6** and **8** (top) and structure of TIPSTAP^10^ for comparison (bottom).

The enlargement of the π‐system from **6** to **8** leads to a significant redshift in both the absorption and emission spectra (Figure 1), the former displaying the typical fine structure of acenes. In comparison with bis(triisopropylsilyl)tetraazapentacene (TIPSTAP; Scheme 1), the *tert*‐butylethynyl substituents of **8** redshifted the absorption by 1612 cm^−1^ from 681 nm to 765 nm.^10^ The first reduction potential of **8** occurs at −0.77 V (−0.81 V for TIPSTAP), whereas the second reduction potential is located at −1.20 V (−1.24 V for TIPSTAP). The similarity of the first and second reduction potentials combined with the considerable redshift suggests that the alkynyl groups primarily lead to a destabilization of the HOMO, but not to a stabilization of the LUMO. This observation is supported by quantum chemical calculations (B3LYP/6‐311++g(d,p)), which display a 0.16 eV destabilization of the LUMO, and a 0.37 eV destabilization of the HOMO, resulting in a calculated decrease of the HOMO–LUMO gap by 0.21 eV (1694 cm^−1^), which corresponds well to the measured difference of 1632 cm^−1^ (Table 1).

**Figure 1:**
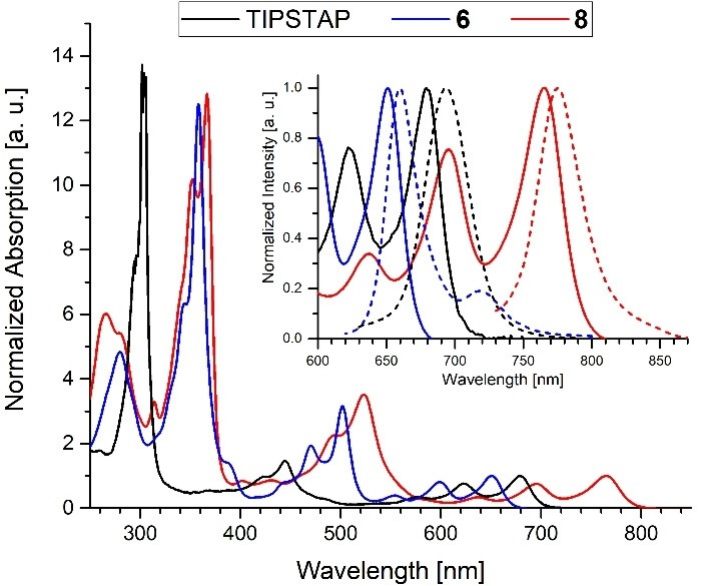
UV/Vis spectra of bis(triisopropylsilyl)tetraazapentacene (TIPSTAP, black) and the peralkynylated derivatives **6** (blue) and **8** (red) in *n*‐hexane. Inset: Normalized absorption and emission spectra magnified to the region of the lowest energy absorption maxima.

**Table 1:** Photophysical and electrochemical properties of TIPSTAP, **6**, and **8**.

Compound	*λ*_max,abs_^[a]^ (*λ*_max,em_)^[a]^ [nm]	*E*_Red_ (0/−1)^[b]^ [V]	*E*_Red_ (−1/−2)^[b]^ [V]	*E*_EA_^[c]^ [eV]	*E*_LUMO_^[d]^ [eV]	*E*_HOMO_^[d]^ [eV]
TIPSTAP	681 (694)	−0.81	−1.24	−3.99	−3.84	−5.58
**6**	651 (660)	−0.76	−1.20	−4.04	−3.72	−5.57
**8**	765 (775)	−0.77	−1.20	−4.03	−3.68	−5.21

[a] Lowest energy absorption and emission maxima recorded in *n*‐hexane at room temperature. [b] First reduction potentials measured by cyclic voltammetry in CH_2_Cl_2_ using Bu_4_NPF_6_ as electrolyte and Fc/Fc^+^ as internal standard (−4.80 eV) at 0.2 V s^−1^.[c] Electron affinity [eV]=−4.8 eV−*E*_Red_(0/−1).^11^ [d] Calculated frontier molecular orbitals by using TURBOMOLE B3LYP/def2‐TZVP//Gaussian 09 B3LYP/6‐311++G**.^12^

Figures 2 and 3 show the single‐crystal X‐ray structures of **6** and **8**. Both arrange in a herringbone‐like structure without π–π contacts to be observed due to the bulky peripheral substituents. The bond lengths are in excellent agreement with the calculated values. Although the tetracene backbone of **6** is only slightly bent, the increased steric pressure upon going to **8** leads to a curious overall geometry, because particularly the central TIPS‐ethynyl groups are highly bent into the opposite directions and the aromatic core is twisted along the long molecular axis, with a maximum of 19° for the central ring. In principle, the presence of the pyrazine rings should have removed all of the *peri* interactions, but the peripheral substituents are too bulky to fit easily. Yet both compounds are stable and can be stored indefinitely in the solid crystalline state.

**Figure 2:**
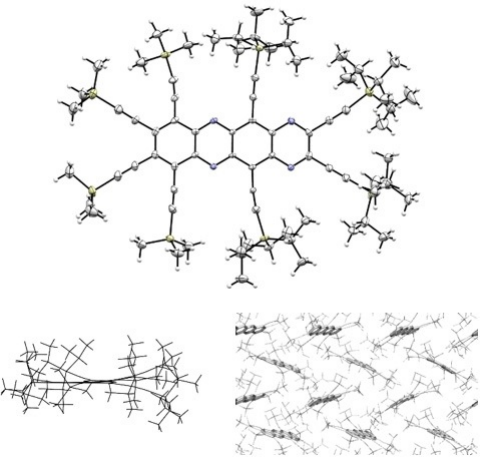
Single‐crystal X‐ray structure of **6**. Ellipsoid plot (top), view from the side (bottom left) and display of the herringbone‐like arrangement (bottom right).

**Figure 3:**
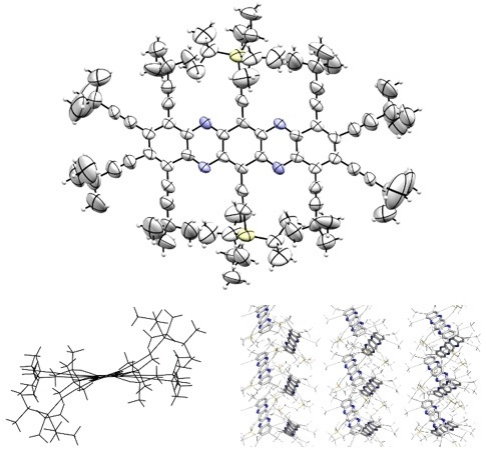
Single‐crystal X‐ray structure of **8**. Ellipsoid plot (top), view from the side (bottom left) and display of the herringbone‐like arrangement (bottom right).

In conclusion, we have prepared the first peralkynylated tetraazatetracene and tetraazapentacene. Despite the excised *peri* interactions—achieved by strategic placement of the pyrazine nitrogen atoms, the alkynes in **8** are bent, and the aromatic perimeter is twisted. The materials are stable and easily processible and should be attractive precursors for pyrolytic transformations into C,N‐type graphene materials. They are stable under ambient conditions, probably due to the steric shielding exerted by the alkyne substituents.

## Experimental Section

CCDC https://www.ccdc.cam.ac.uk/services/strctures?id=doi:10.1002/chem.201904087 contain the supplementary crystallographic data for this paper. These data are provided free of charge by http://www.ccdc.cam.ac.uk/. The synthetic details for precursors and remaining materials can be found in the Supporting Information.

**2,3,5,12‐Tetrakis((triisopropylsilyl)ethynyl)‐7,8,9,10‐tetrakis((trimethylsilyl)ethynyl)‐1,2‐dihydropyrazino[2,3‐*b*]phenazine (6)**: **4 a** (43.0 mg, 45.0 μmol, 1 equiv) and **5** (24.5 mg, 58.5 μmol, 1.3 equiv) were stirred for 6 h in 2.00 mL of DCM/AcOH (1:1) at room temperature. The mixture was poured into DI water and extracted with DCM. The organic phases were treated with sat. aqueous sodium bicarbonate and dried over magnesium sulfate. After evaporation of the solvent under reduced pressure, the crude product was purified by chromatography on silica using PE/DCM 9:1→6:1→4:1 as eluent to give the product as a turquoise solid (37.0 mg, 27.6 μmol, 61 %). M.p.: >400 °C. ^1^H NMR (600 MHz, CDCl_3_): *δ* [ppm]=1.25–1.23 (m, 42 H), 1.21–1.18 (m, 42 H), 0.37 (s, 18 H), 0.37 (s, 18 H). ^13^C {^1^H} NMR (150 MHz, CDCl_3_): *δ* [ppm]=143.1, 142.2, 142.1, 141.6, 131.9, 126.8, 122.3, 113.4, 110.4, 109.8, 103.9, 102.4, 102.0, 101.9, 100.9, 19.2, 18.9, 11.9, 11.8, 0.6, 0.3. IR (neat): $\tilde{\nu }$ [cm^−1^]=2942, 2891, 2863, 1461, 1442, 1384, 1325, 1246, 1163, 1108, 1041, 944, 836, 793, 757, 675, 643, 607, 578, 489, 405. HR‐MS(MALDI+): *m*/*z* calcd for C_78_H_121_N_4_Si_8_: 1337.7740; found: 1337.7965. Elemental analysis (%) calcd for C_78_H_120_N_4_Si_8_: C 69.99, H 9.04, N 4.19; found: C 69.73, H 8.92, N 3.55. UV/Vis: *λ*_max, abs_ (hexane)=651 nm. Fluorescence: *λ*_max,em_ (hexane)=660 nm.

**1,2,3,4‐Tetrabromo‐8,9,10,11‐tetrakis(3,3‐dimethylbut‐1‐yn‐1‐yl)‐6,13‐bis((triisopropylsilyl)ethynyl)‐5,14‐dihydroquinoxalino[2,3‐*b*]phenazine (7 b‐H_2_)**: In a heatgun‐dried Schlenk tube under argon with molecular sieves, **4 b** (50.0 mg, 56.1 μmol, 1.0 equiv) and tetrabromoorthoquinone (47.5 mg, 112 μmol, 2.0 equiv) were dissolved in 2 mL of abs. toluene and 0.50 mL AcOH. The reaction mixture stirred for 2 days at room temperature while it turned from red to brown. After completion, checked via TLC, the mixture was filtered through Celite. Water was added, and the phases were separated; DCM was used for extraction. The organic phases were treated with sat. aqueous sodium bicarbonate and dried over magnesium sulfate. After evaporation of the solvent, the crude product was purified by chromatography on silica by using PE/DCM 10:1>5:1 as eluent to give a mixture of the oxidized and reduced form of **7 b**. It was dissolved with 5.00 mL THF and stirred with SnCl_2_ (106 mg, 561 μmol, 10 equiv) in 0.50 mL conc. HCl for 30 min. Water was added to the blue solution, and the phases were separated; DCM was used for extraction. The organic phases were treated with sat. aqueous sodium bicarbonate and dried over magnesium sulfate. After evaporation of the solvent, the crude product was purified by chromatography on silica using PE/DCM 10:1→5:1 to give the product as a purple solid (6.00 mg, 4.69 μmol, 8 %). ^1^H NMR (600 MHz, CDCl_3_): *δ* [ppm]=7.65 (s, 2 H), 1.41 (s, 18 H), 1.39 (s, 18 H), 1.20–1.17 (m, 42 H). ^13^C {^1^H} NMR (150 MHz, CDCl_3_): *δ* [ppm]=146.1, 139.4, 139.2, 128.0, 127.7, 127.4, 124.0, 110.9, 110.4, 106.6, 105.6, 99.0, 97.7, 72.3, 31.5, 31.5, 29.9, 29.8, 29.5, 19.2, 12.2. IR (neat): $\tilde{\nu }$ [cm^−1^]=3347, 2921, 2859, 1678, 1592, 1570, 1444, 1404, 1385, 1238, 1176, 1102, 1049, 1009, 975, 882, 786, 675, 606, 559, 463. HR‐MS(MALDI+): *m*/*z* calcd for C_64_H_81_Br_4_N_4_Si_2_: 1277.2728; found: 1277.2694. UV/Vis: *λ*_max,abs_ (hexane)=591 nm.

**1,2,3,4,8,9,10,11‐Octakis(3,3‐dimethylbut‐1‐yn‐1‐yl)‐6,13‐bis((triisopropylsilyl)ethynyl)‐5,14‐dihydroquinoxalino[2,3‐*b*]phenazine 8‐H_2_**: Bis(benzonitrile)palladium(II) dichloride (1.00 mg, 2.61 μmol, 0.28 equiv) was placed in a Schlenk tube and transferred into a glovebox under nitrogen atmosphere. Absolute toluene (2.00 mL) was added, followed by P(*t*Bu)_3_ (1.06 mg, 5.25 μmol, 0.56 equiv). The Schlenk tube was closed and taken outside the glovebox. Then, against a counterflow of argon, **7 b‐H_2_** (12.0 mg, 9.37 μmol, 1 equiv) and (3,3‐dimethylbut‐1‐yn‐1‐yl)trimethylstannane (27.5 mg, 112.4 μmol, 12 equiv) were added, and the reaction mixture was stirred at 90 °C for 16 h. After adding DI water, extraction with DCM, and drying of the organic phases over magnesium sulfate, the crude mixture was filtered through a pad of silica. The solvent was evaporated under reduced pressure, and the product was purified by chromatography on silica by using PE/DCM 9:1→6:1 to give the product as a purple red solid (8.00 mg, 6.22 μmol, 66 %). ^1^H NMR (600 MHz, CDCl_3_): *δ* [ppm]=7.53 (s, 2 H), 1.44 (s, 18 H), 1.42 (s, 18 H), 1.40 (s, 18 H), 1.38 (s, 18 H), 1.13–1.09 (m, 42 H). ^13^C {^1^H} NMR (150 MHz, CDCl_3_): *δ* [ppm]=143.5, 141.1, 138.4, 128.4, 128.3, 124.8, 123.1, 110.1, 109.8, 109.5, 107.4, 104.8, 104.4, 101.4, 98.4, 78.5, 77.1, 76.7, 31.5, 31.5, 31.5, 31.5, 28.8, 28.8, 28.6, 19.2, 11.5. IR (neat): $\tilde{\nu }$ [cm^−1^]=3354, 2957, 2923, 2861, 2359, 2342, 1726, 1571, 1454, 1246, 1017, 798, 658, 556, 452. HR‐MS(MALDI+): *m*/*z* calcd for C_88_H_117_N_4_Si_2_: 1285.8811; found: 1285.8785. UV/Vis: *λ*_max,abs_ (hexane)=587 nm. Fluorescence: *λ*_max,em_ (hexane)=596 nm.

**1,2,3,4,8,9,10,11‐Octakis(3,3‐dimethylbut‐1‐yn‐1‐yl)‐6,13‐bis((triisopropylsilyl)ethynyl)‐quinoxalino[2,3‐*b*]phenazine (8)**: **8‐H_2_** (5.00 mg, 3.89 μmol, 1 equiv) was dissolved in 3.00 mL DCM and treated with MnO_2_ (3.38 mg, 38.9 μmol, 10 equiv). The reaction mixture quickly turned from purple to red brown. After 30 min and complete conversion of the starting material, the reaction mixture was filtered through Celite, and the solvent was evaporated under reduced pressure. Chromatography on silica using PE/DCM 5:1→2:1 as eluent gave the product as a red solid (4.80 mg, 3.74 μmol, 96 %). M.p.: 270 °C (decomp.) ^1^H NMR (600 MHz, CDCl_3_): *δ* [ppm]=1.48–1.47 (m, 72 H), 1.16–1.13 (m, 42 H). ^13^C {^1^H} NMR (150 MHz, CDCl_3_): *δ* [ppm]=143.5, 142.4, 131.2, 125.8, 122.3, 115.3, 111.9, 111.6, 104.9, 78.9, 76.4, 31.4, 31.3, 29.1, 29.0, 19.2, 11.7. IR (neat): $\tilde{\nu }$ [cm^−1^]=2965, 2922, 2862, 2360, 2341, 2214, 1445, 1396, 1361, 1238, 1112, 1061, 802, 727, 659, 587. HR‐MS(MALDI+): *m*/*z* calcd for C_88_H_115_N_4_Si_2_: 1283.8655; found: 1283.8691. UV/Vis: *λ*_max,abs_ (hexane)=765 nm. Fluorescence: *λ*_max,em_ (hexane)=775 nm.

## Conflict of interest

The authors declare no conflict of interest.

## Supporting information

As a service to our authors and readers, this journal provides supporting information supplied by the authors. Such materials are peer reviewed and may be re‐organized for online delivery, but are not copy‐edited or typeset. Technical support issues arising from supporting information (other than missing files) should be addressed to the authors.

Supplementary
