# Supplementary material for: Peralkynylated Tetraazaacene Derivatives
Source: Chemistry. 2019 Dec 16;26(5):1013–6. doi: 10.1002/chem.201904087 (PMC7028064; doi:10.1002/chem.201904087)
Supplement: Supplementary file 1 — Supplementary [file CHEM-26-1013-s001.pdf]

CHEMISTRY
A European Journal

Supporting Information

Peralkynylated Tetraazaacene Derivatives

Hilmar Reiss,[a] Frank Rominger,[a] Jan Freudenberg,[a, c] and Uwe H. F. Bunz*[a, b]

chem_201904087_sm_miscellaneous_information.pdf

Table of Contents

**Experimental Procedures**	**2**
**Optical Spectroscopy**	**7**
**Cyclic Voltammetry**	**11**
**Calculations**	**12**
**NMR-Spectra**	**13**
**IR-Spectra**	**25**
**Crystallographic Data**	**32**
**References**	**34**

Experimental Procedures

General Procedures, General Purification

Reagents and solvents were obtained from commercial suppliers and used without further purification. For chromatographic purifications, manual column chromatography (SiO2, grain size of 0.04-0.063 mm) was used. NMR spectra were recorded on Bruker Avance Spectrometers using the specified frequency. Chemical shifts (δ) are given in parts per million (ppm) relative to internal solvent signals.[1] The following abbreviations describe the signal multiplicities: s = singlet, m=multiplet. High-resolution mass spectra (HRMS) were obtained by matrix-assisted laser desorption/ionization (MALDI), electrospray ionisation (ESI) or direct analysis in real time (DART) experiments. IR Spectra were recorded from powder of the respective analyte on a Jasco FT/IR-4100 spectrometer. CV measurements were performed on a VersaSTAT 3 potentiostat by Princeton Applied Research. UV-vis spectra were recorded on a Jasco V670. Computational studies were carried out using DFT calculations on Turbomole 6.3.1 or Gaussian 09. Geometry optimizations were performed using the B3LYP functional and def2-TZVP basis set. At this geometry, the absolute energy and FMO energies were assigned by a single-point approach at the B3LYP/6–311++G** level.[2]

Synthetic procedures

Procedure for stannylacetylenes: The stannylacetylenes were prepared according to the literature:[3] In a heat-gun dried Schlenk tube under argon atmosphere the alkyne (1 equiv.) was dissolved in abs. Et_2O and cooled to −78 °C before it was treated with 1.05 equiv. of *n*-BuLi (2.5 M in hexane). The reaction was stirred for 1 h at this temperature then 1.10 equiv. of Me_3SnCl (1 M in THF) were added. Stirring over night at room temperature followed by aqueous workup, extraction with Et_2O, drying with magnesium sulfate and removal of the solvent gave the stannylacetylene, which was used without further purification.

General Procedure 1 (GP1): The N,N'-dihydroazaacene was dissolved in DCM and 10 equiv. of MnO_2 were added. After complete conversion of the starting material the reaction mixture was filtered through Celite and the solvent was evaporated under reduced pressure. The crude product was purified by column chromatography.

General Procedure 2 (GP2): In a heat-gun dried Schlenk tube under argon atmosphere the thiadiazolophenazine derivative was dissolved in abs. THF / abs. MeOH (40:1). At −10 °C SmI_2 (0.1 M in THF, 12 equiv.) was added dropwise. After 10 minutes at this temperature the mixture was poured into sat. aqueous NaCl solution. Et_2O was used for extraction. The combined organic phases

were washed with sat. aqueous NaS_2O_3 solution, then dried over magnesium sulfate. After evaporation of the solvent under reduced pressure, the crude product was purified by column chromatography.

6,7,8,9-Tetrabromo-4,11-bis((triisopropylsilyl)ethynyl)-[1,2,5]thiadiazolo[3,4-b]phenazine 2

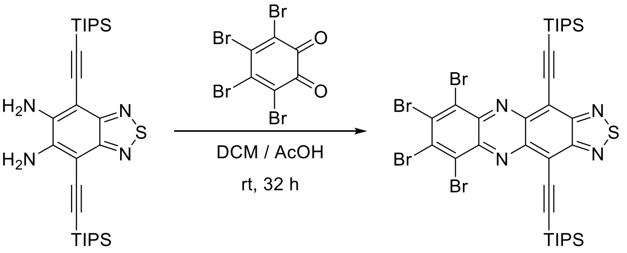

1 **2**, 75%

1 (250 mg, 474 µmol, 1 equiv.) and tetrabromoorthoquinone (241 mg, 569 µmol, 1.2 equiv.) were stirred for 32 h in 5.00 mL of DCM / AcOH (1:1) at room temperature. The mixture was poured into DI water and extracted with DCM. The organic phases were treated with sat. aqueous sodium bicarbonate and dried over magnesium sulfate. After evaporation of the solvent under reduced pressure, the crude product was purified by chromatography on silica using PE / DCM 9:1 -> 5:1 as eluent to give the product as a dark green crystalline solid (326 mg, 356 µmol, 75 %). M.p.: 258 °C. ^{1}H NMR (500 MHz, $CDCl_3$): δ [ppm] = 1.31-1.28 (m, 42H). ^{13}C {^{1}H} NMR (100 MHz, $CDCl_3$): δ [ppm] = 156.0, 142.3, 141.3, 133.1, 129.0, 115.2, 113.9, 101.7, 19.1, 11.8. IR (neat): ν [cm^{-1}] = 2939, 2861, 1550, 1442, 1402, 1363, 1339, 1231, 1186, 1156, 1132, 1067, 1042, 1026, 994, 941, 918, 881, 854, 811, 754, 670, 612, 566, 535, 507, 489, 452, 415. HR-MS (ESI-): m/z calcd. for $C_{34}H_{42}Br_4N_4SSi_2$: 909.9408; found: 909.9423. UV-Vis: $\lambda_{max,\ abs}$ (hexane) = 673 nm.

4,11-Bis((triisopropylsilyl)ethynyl)-6,7,8,9-tetrakis((trimethylsilyl)ethynyl)-5,10-dihydro-[1,2,5]thiadiazolo[3,4-b]phenazine 3a-H2

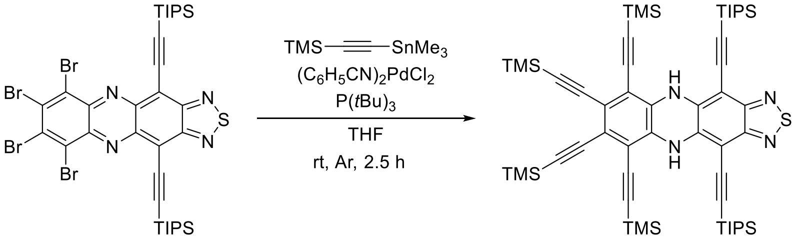

2 **3a-H$_2$**, 79%

Bis(benzonitrile)palladium(II) dichloride (16.8 mg, 43.7 µmol, 0.1 equiv.) was placed in a Schlenk tube and transferred into a glove-box under nitrogen atmosphere. 10.0 mL abs. THF were added followed by P(*t*-Bu)$_3$ (17.7 mg, 87.5 µmol, 0.2 equiv.). The Schlenk tube was closed and taken outside the glove-box. Then, against a counter-flow of argon, **2** (400 mg, 437 µmol, 1 equiv.) and trimethyl((trimethylstannyl)-ethynyl)silane (1.37 g, 5.25 mmol, 12 equiv.) were added and the reaction mixture stirred at room temperature for 2.5 h. After adding DI water, extraction with DCM and drying of the organic phases over magnesium sulfate, the crude solution was filtered through a pad of silica. The solvent was evaporated under reduced pressure and the crude product was purified by chromatography on silica using PE / DCM 20:1 -> 10:1 -> 5:1 as eluent to give the product as a red solid (340 mg, 345 µmol, 79%). M.p.: 280 °C decomp.. ^{1}H NMR (600 MHz, $CDCl_3$): δ [ppm] = 7.23 (s, 2H), 1.19-1.17 (m, 42H), 0.31 (s, 18H), 0.28 (s, 18H). ^{13}C {^{1}H} NMR (150 MHz, $CDCl_3$): δ [ppm] = 153.1, 136.2, 129.5, 123.4, 109.1, 108.3, 106.7, 102.2, 101.6, 98.1, 97.6, 94.4, 19.0, 11.7, 0.5, 0.5. IR (neat): ν [cm^{-1}] = 3355, 2942, 2864, 2146, 1563, 1457, 1405, 1374, 1317, 1280, 1246, 1040, 929, 881, 838, 757, 698, 669, 640, 574, 545, 515. HR-MS(DART+): m/z calcd. for $C_{54}H_{81}N_4SSi_6$: 985.4792; found: 985.4813. Elemental analysis (%) calcd. for $C_{54}H_{80}N_4SSi_6$: C 65.79, H 8.18, N 5.68, found: C 64.62, H 8.29, N 5.24. UV-Vis: $\lambda_{max,\ abs}$ (hexane) = 530 nm. Fluorescence: $\lambda_{max,\ em}$ (hexane) = 541 nm.

***4,11-Bis((triisopropylsilyl)ethynyl)-6,7,8,9-tetrakis((trimethylsilyl)ethynyl)-[1,2,5]thiadiazolo[3,4-b]phenazine* 3a**

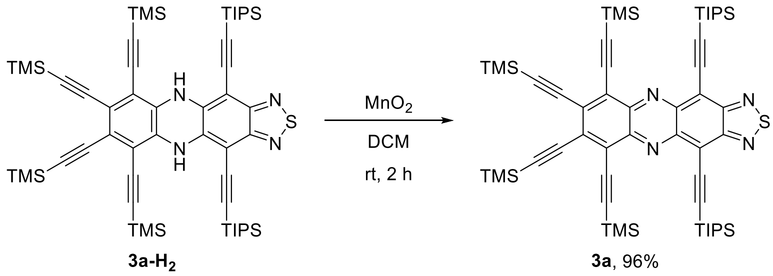

GP1 was applied to **3a-H$_2$** (300 mg, 304 µmol, 1 equiv.) and MnO_2 (265 mg, 3.04 mmol, 10 equiv.) in 20.0 mL DCM. The reaction mixture quickly turned from red to green. After 2 h at room temperature the reaction was worked up. Chromatography on silica using PE / DCM 10:1 -> 5:1 -> 3:1 as eluent gave the product as a green solid (287 mg, 292 µmol, 96%). M.p.: 265 °C. ^{1}H NMR (600 MHz, $CDCl_3$): δ [ppm] = 1.29-1.25 (m, 42H), 0.39 (s, 18H), 0.38 (s, 18H). ^{13}C {^{1}H} NMR (150 MHz, $CDCl_3$): δ [ppm] = 156.4, 143.5, 141.3, 132.1, 126.8, 115.2, 113.7, 110.5, 102.6, 102.3, 100.6, 19.2, 11.8, 0.6, 0.3. IR (neat): ν [cm^{-1}] = 2942, 2863, 1737, 1449, 1381, 1364, 1246, 1198, 1109, 1040, 937, 916, 837, 757, 700, 675, 641, 575, 405. HR-MS(DART+): m/z calcd. for $C_{54}H_{79}N_4SSi_6$: 983.4636; found: 983.4624. UV-Vis: $\lambda_{max,\ abs}$ (hexane) = 693 nm. Fluorescence: $\lambda_{max,\ em}$ (hexane) = 711 nm.

***6,7,8,9-Tetrakis(3,3-dimethylbut-1-yn-1-yl)-4,11-bis((triisopropylsilyl)ethynyl)-5,10-dihydro-[1,2,5]thiadiazolo[3,4-b]phenazine* 3b-H$_2$**

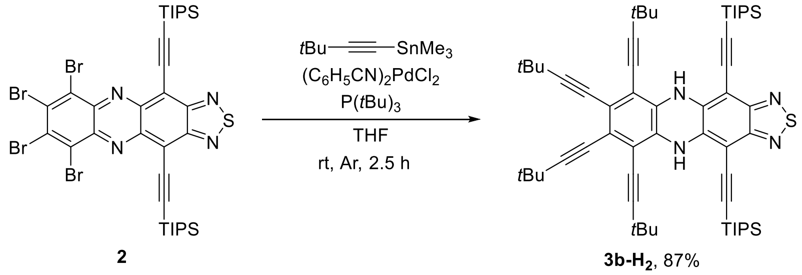

Bis(benzonitrile)palladium(II) dichloride (16.8 mg, 43.7 µmol, 0.1 equiv.) was placed in a Schlenk tube and transferred into a glove-box under nitrogen atmosphere. 10.0 mL abs. THF were added followed by P(*t*-Bu)$_3$ (17.7 mg, 87.5 µmol, 0.2 equiv.). The Schlenk tube was closed and taken outside the glove-box. Then, against a counter-flow of argon, **2** (400 mg, 437 µmol, 1 equiv.) and (3,3-dimethylbut-1-yn-1-yl)trimethylstannane (1.29 g, 5.25 mmol, 12 equiv.) were added and the reaction mixture stirred at room temperature for 2.5 h. After adding DI water, extraction with DCM and drying of the organic phases over magnesium sulfate, the crude solution was filtered through a pad of silica. The solvent was evaporated under reduced pressure and the crude product was purified by chromatography on silica using PE / DCM 9:1 -> 6:1 -> 4:1 as eluent to give the product as a red solid (349 mg, 378 µmol, 87%). M.p.: 298 °C decomp. ^{1}H NMR (600 MHz, $CDCl_3$): δ [ppm] = 7.15 (s, 2H), 1.41(s, 18H), 1.37 (s, 18H), 1.19-1.17 (m, 42H). ^{13}C {^{1}H} NMR (150 MHz, $CDCl_3$): δ [ppm] = 153.4, 136.7, 128.1, 122.7, 110.2, 109.3, 106.3, 104.7, 99.0, 93.5, 72.4, 31.6, 31.5, 28.7, 28.6, 19.0, 11.8. IR (neat): ν [cm^{-1}] = 3356, 2965, 2942, 2863, 2215, 2127, 1561, 1453, 1379, 1359, 1317, 1277, 1239, 1202, 1000, 880 ,834, 814, 769, 674, 643, 576, 548, 512, 464. HR-MS(DART+): m/z calcd. for $C_{58}H_{81}N_4SSi_2$: 921.5715; found: 921.5703. UV-Vis: $\lambda_{max,\ abs}$ (hexane) = 528 nm. Fluorescence: $\lambda_{max,\ em}$ (hexane) = 532 nm.

***6,7,8,9-Tetrakis(3,3-dimethylbut-1-yn-1-yl)-4,11-bis((triisopropylsilyl)ethynyl)-[1,2,5]thiadiazolo[3,4-b]phenazine* 3b**

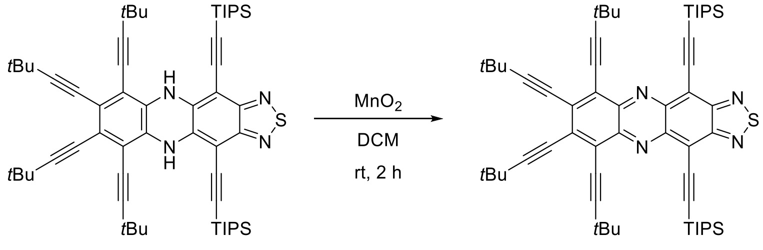

GP1 was applied to **3b-H$_2$** (500 mg, 543 µmol, 1 equiv.) and MnO_2 (471 mg, 5.43 mmol, 10 equiv.) in 30.0 mL DCM. The reaction mixture quickly turned from red to green. After 2 h at room temperature the reaction was worked up. Chromatography on silica using PE / DCM 10:1 -> 5:1 -> 3:1 as eluent gave the product as a green solid (490 mg, 533 µmol, 98%). M.p.: 273 °C. ^{1}H NMR (600 MHz, $CDCl_3$): δ [ppm] = 1.52 (s, 18H), 1.47 (s, 18H), 1.28-1.24 (m, 42H). ^{13}C {^{1}H} NMR (150 MHz, $CDCl_3$): δ [ppm] = 156.5, 144.3, 141.1, 131.7, 125.8, 115.1, 112.9, 112.3, 112.0, 103.3, 78.8, 76.0, 31.5, 31.3, 29.1, 29.0, 19.1, 11.9. IR (neat): ν [cm^{-1}] = 2964, 2941, 2863, 2209, 1452, 1391, 1360, 1238, 1201, 1121, 1052, 1002, 90, 881, 780, 702, 674, 580, 443, 409. HR-MS(DART+): m/z calcd. for $C_{58}H_{79}N_4SSi_2$: 919.5558; found: 919.5539. UV-Vis: $\lambda_{max, abs}$ (hexane) = 692 nm. Fluorescence: $\lambda_{max, em}$ (hexane) = 700 nm.

***1,4-Bis((triisopropylsilyl)ethynyl)-6,7,8,9-tetrakis((trimethylsilyl)ethynyl)phenazine-2,3-diamine* 4a**

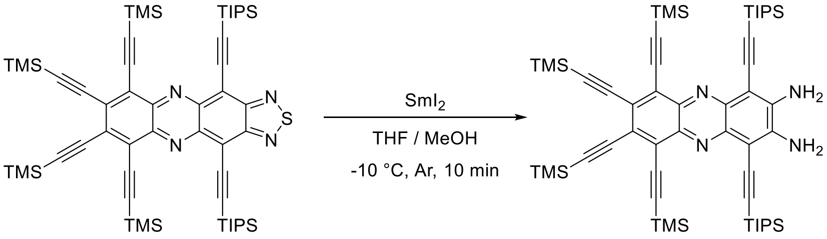

GP2 was applied to **3a** (230 mg, 234 µmol, 1 equiv.) and SmI_2 (28.1 mL, 2.81 mmol, 0.1 M in THF, 12 equiv.) in 10.0 mL abs. THF and 0.25 mL abs. MeOH. After workup the crude product was purified by chromatography on silica using PE / EE 20:1 -> 10:1 -> 5:1 as eluent to give the product as an orange solid (222 mg, 232 µmol, quant.). M.p.: 236. ^{1}H NMR (600 MHz, $CDCl_3$): δ [ppm] = 5.06 (s, 4H), 1.22-1.19 (m, 42H), 0.36 (s, 18H), 0.34 (s, 18H). ^{13}C {^{1}H} NMR (150 MHz, $CDCl_3$): δ [ppm] = 145.4, 140.9, 140.4, 129.2, 126.0, 108.0, 105.8, 104.6, 102.7, 102.6, 101.6, 101.2, 19.1, 11.7, 0.7, 0.5. IR (neat): ν [cm^{-1}] = 3459, 3369, 2940, 2863, 2242, 2130, 1646, 1529, 1442, 1385, 1317, 882, 756, 662. HR-MS(DART+): m/z calcd. for $C_{54}H_{79}N_4SSi_6$: 955.5228; found: 955.5235.

***6,7,8,9-Tetrakis(3,3-dimethylbut-1-yn-1-yl)-1,4-bis((triisopropylsilyl)ethynyl)phenazine-2,3-diamine* 4b**

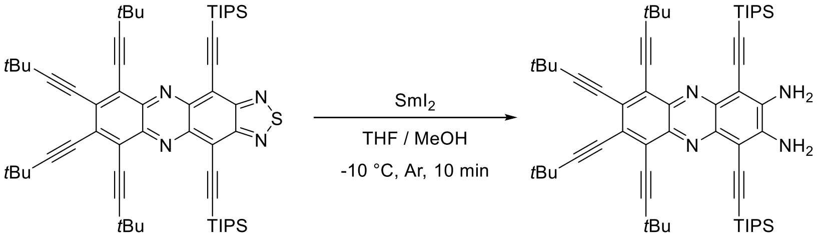

GP2 was applied to **3b** (240 mg, 261 µmol, 1 equiv.) and SmI_2 (31.3 mL, 3.13 mmol, 0.1 M in THF, 12 equiv.) in 10.0 mL abs. THF and 0.25 mL abs. MeOH. After workup the crude product was purified by chromatography on silica using PE / EE 20:1 -> 10:1 as eluent to give the product as an orange solid (231 mg, 259 µmol, quant.). M.p.: 249 °C. ^{1}H NMR (600 MHz, $CDCl_3$): δ [ppm] =4.96 (s, 4H), 1.49 (s, 18H), 1.45 (s, 18H), 1.21-1.18 (m, 42H). ^{13}C {^{1}H} NMR 150 MHz, $CDCl_3$): δ [ppm] = 145.2, 140.8, 140.2, 128.7, 125.2, 109.8, 107.9, 104.1, 103.2, 102.1, 78.5, 76.5, 31.6, 31.5, 28.9, 28.8, 19.1, 11.8. IR (neat): ν [cm^{-1}] = 3455, 3368, 2965, 2863, 2219, 2124, 1624,

1538, 1504, 1448, 1412, 1360, 1244, 1202, 1133, 1102, 995, 970, 882, 803, 675, 580, 445, 425. HR-MS(DART+): m/z calcd. for $C_{58}H_{83}N_4Si_2$: 891.6151; found: 891.6143.

1,2,3,4-Tetrabromo-6,13-bis((triisopropylsilyl)ethynyl)-8,9,10,11-tetrakis((trimethylsilyl)ethynyl)-quinoxalino[2,3-b]phenazine 7a

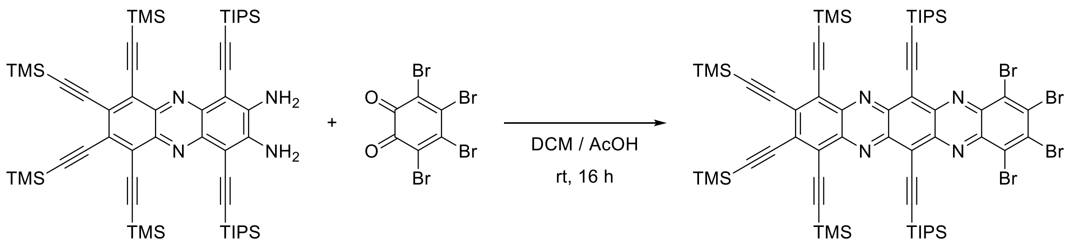

4a **7a**, 5%

4a (56.0 mg, 58.6 µmol, 1 equiv.) and tetrabromoorthoquinone (49.7 mg, 117 µmol, 2 equiv.) were stirred for 16 h in 4.00 mL of DCM / AcOH (1:1) at room temperature. The mixture was poured into DI water and extracted with DCM. The organic phases were treated with sat. aqueous sodium bicarbonate and dried over magnesium sulfate. After evaporation of the solvent under reduced pressure, the crude product was purified by chromatography on silica using PE / DCM 20:1 -> 10:1 -> 5:1 as eluent to give the product as a brown solid (4.00 mg, 2.98 µmol, 5%). ^{1}H NMR (600 MHz, $CDCl_3$): δ [ppm] = 1.30-1.26 (m, 42H), 0.38 (s, 18H), 0.38 (s, 18H). ^{13}C {^{1}H} NMR (150 MHz, $CDCl_3$): δ [ppm] = 143.7, 143.6, 142.9, 141.3, 133.0, 132.6, 129.1, 126.8, 123.5, 116.3, 111.0, 111.0, 102.8, 102.4, 100.9, 19.3, 12.0, 0.6, 0.3. IR (neat): ν [cm^{-1}] = 3460 3369, 2959, 2942, 2864, 2359, 2340, 2145, 1647, 1625, 1455, 1247, 1128, 1025, 935, 844, 759, 678, 580. HR-MS(MALDI+): m/z calcd. for $C_{54}H_{79}N_4SSi_6$: 1339.1648; found: 1339.1693. UV-Vis: λ_{max} (hexane) = 769 nm.

Optical Spectroscopy

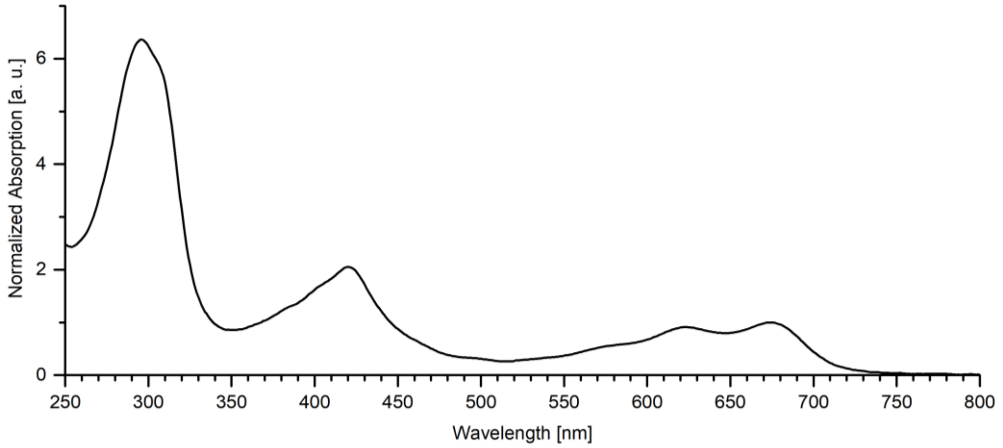

Figure S1. Normalized Absorption Spectrum of **2**.

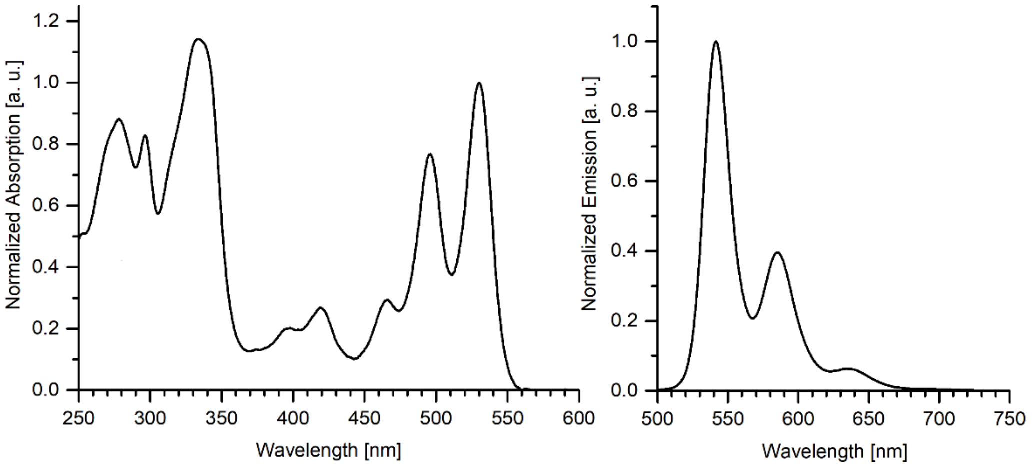

Figure S2. Normalized Absorption (left) and Emission (right) Spectra of **3a-H$_2$**.

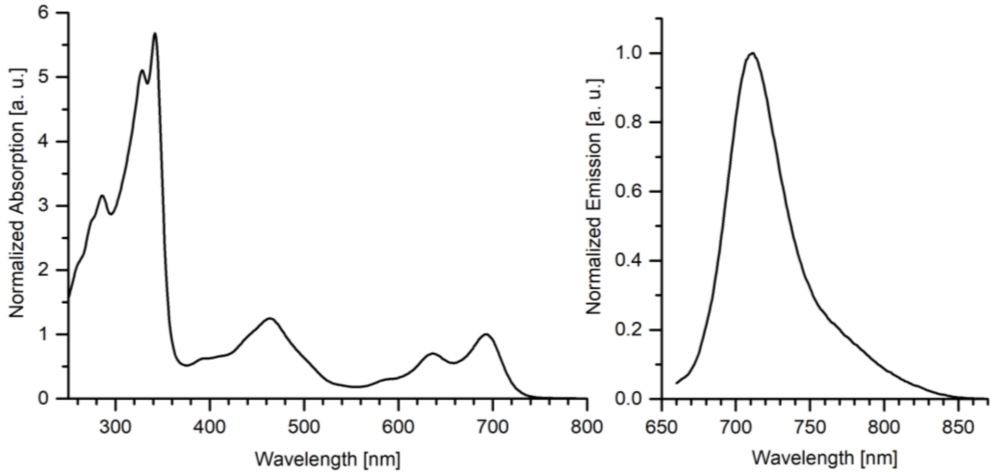

Figure S3. Normalized Absorption (left) and Emission (right) Spectra of **3a**.

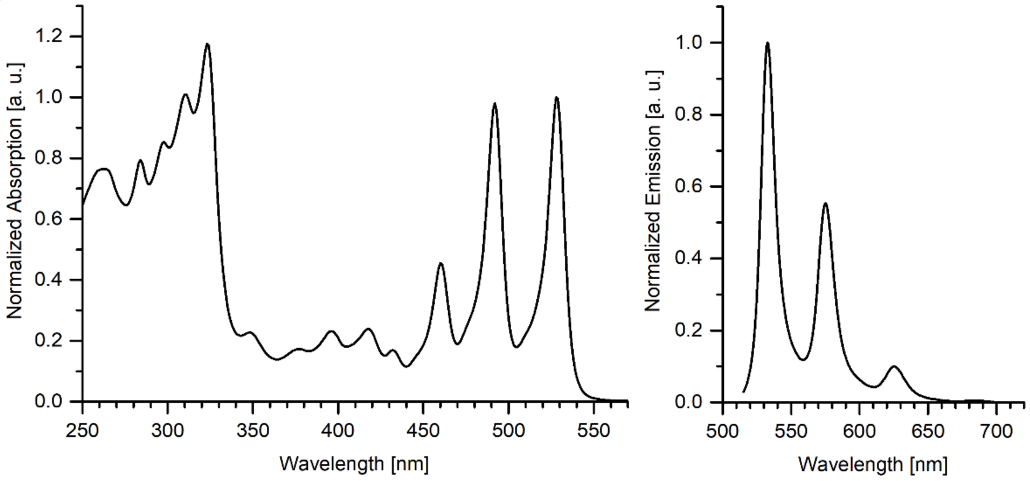

Figure S4. Normalized Absorption (left) and Emission (right) Spectra of **3b-H_2**.

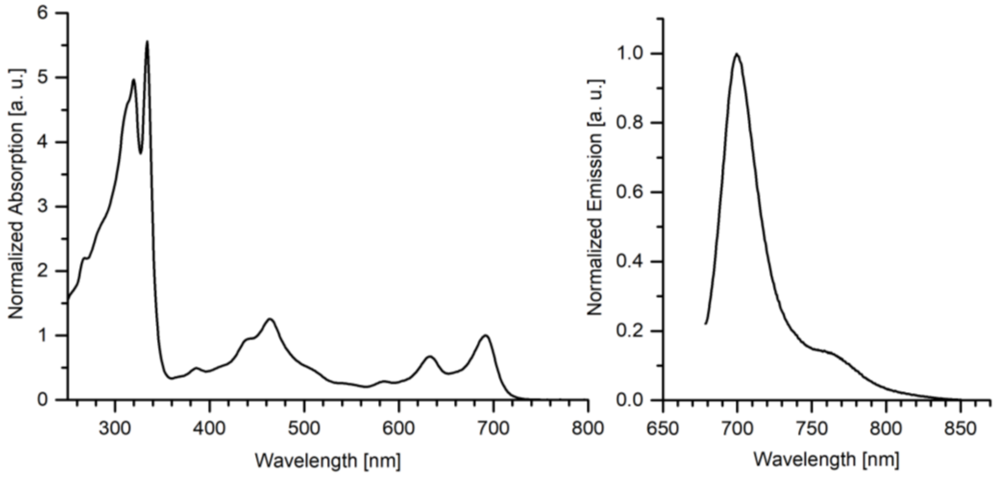

Figure S5. Normalized Absorption (left) and Emission (right) Spectra of **3b**.

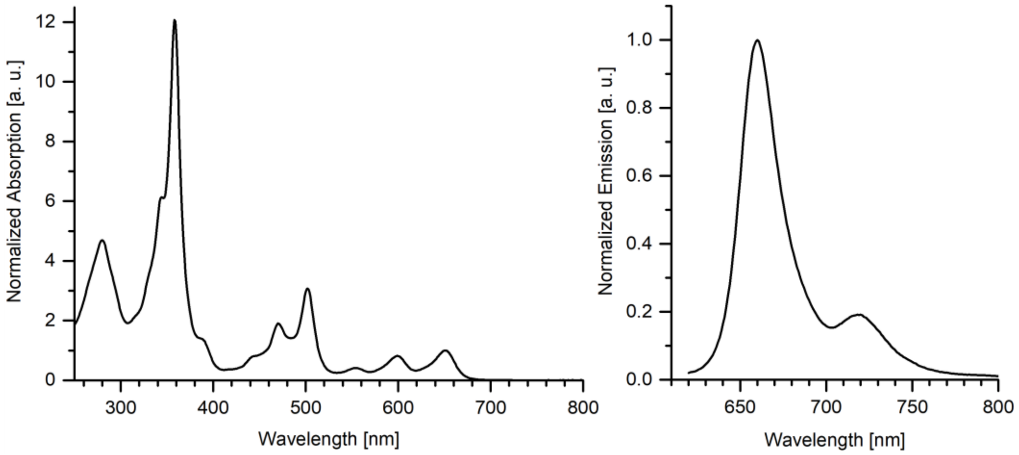

Figure S6. Normalized Absorption (left) and Emission (right) Spectra of **6**.

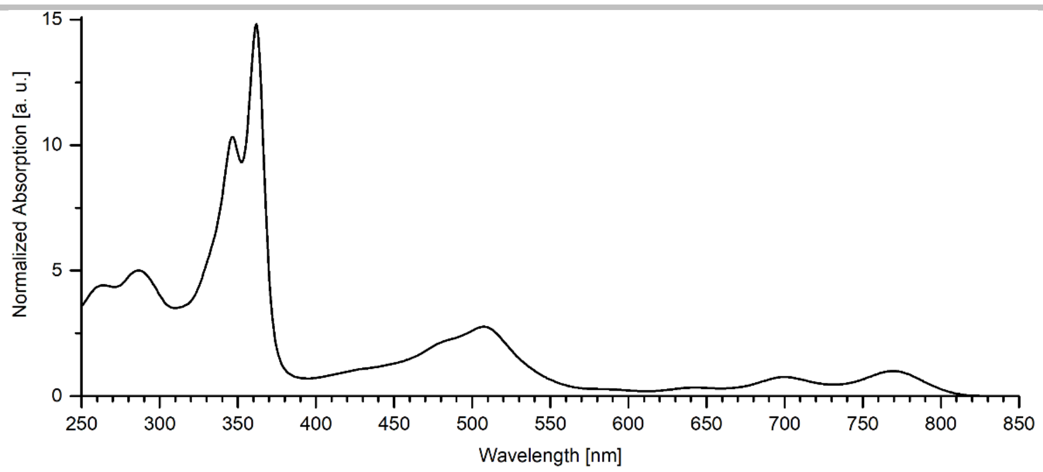

Figure S7. Normalized Absorption Spectrum of **7a**.

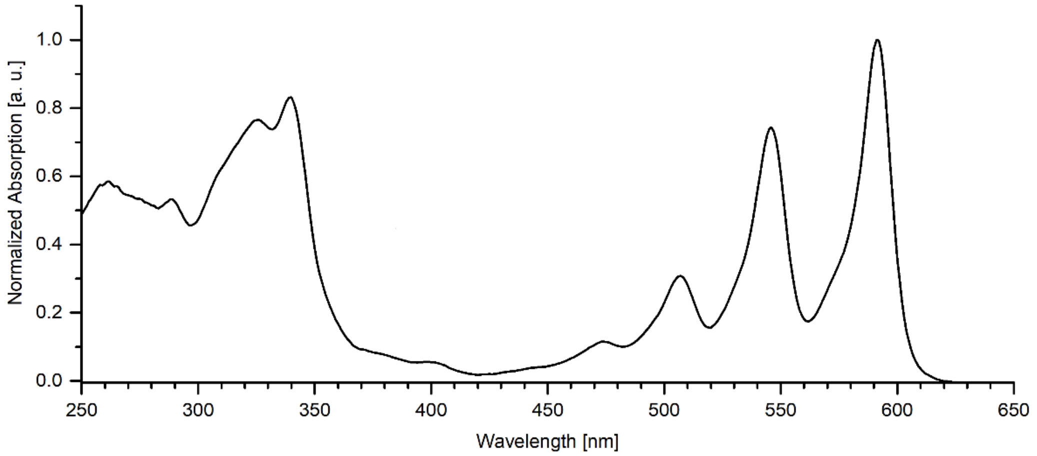

Figure S8. Normalized Absorption Spectrum of **7b-H$_2$**.

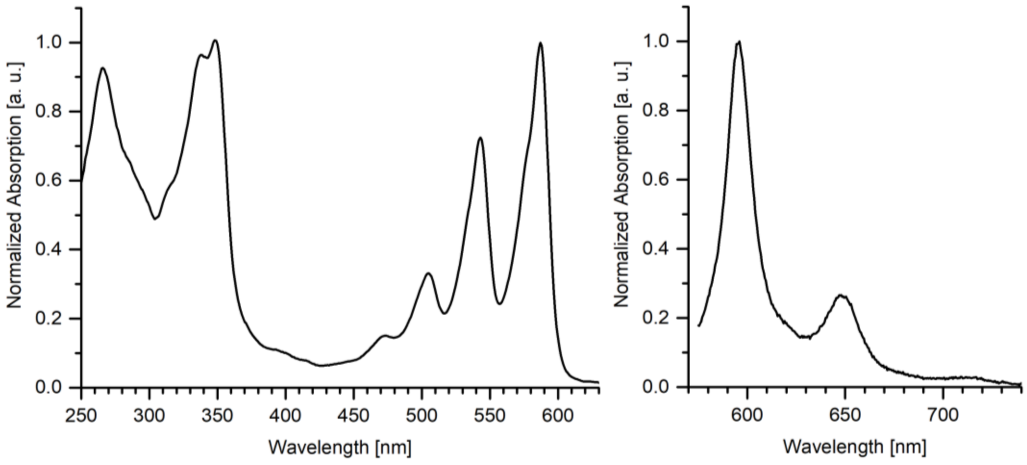

Figure S9. Normalized Absorption (left) and Emission (right) Spectra of **8-H$_2$**.

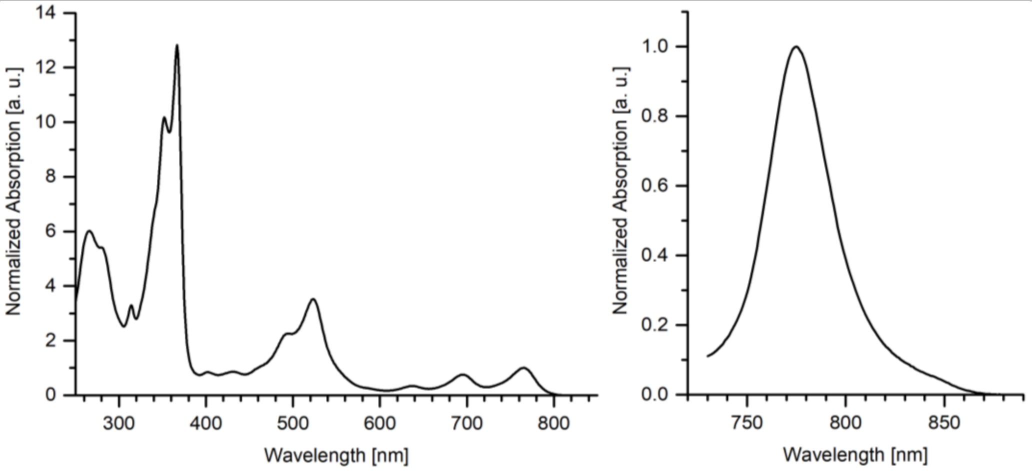

Figure S10. Normalized Absorption (left) and Emission (right) Spectra of **8**.

Cyclic Voltammetry

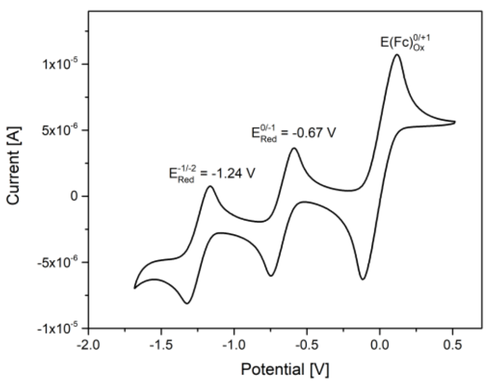

Figure S11. Cyclic voltammogram of **2**.

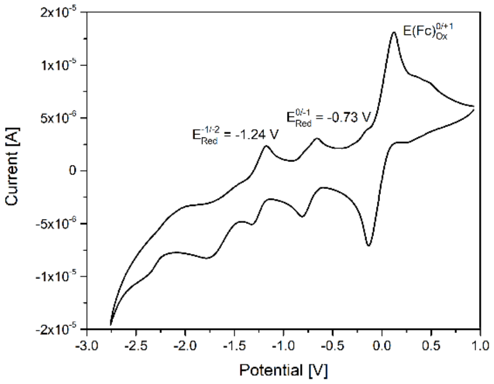

Figure S12. Cyclic voltammogram of **3a**.

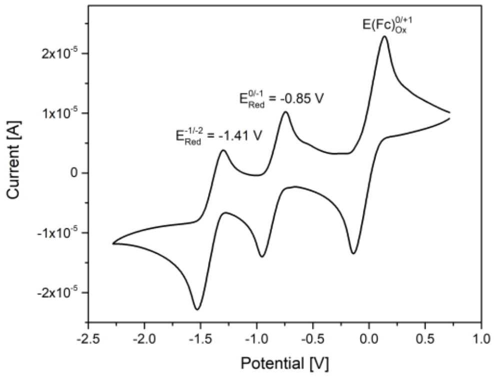

Figure S13. Cyclic voltammogram of **3b**.

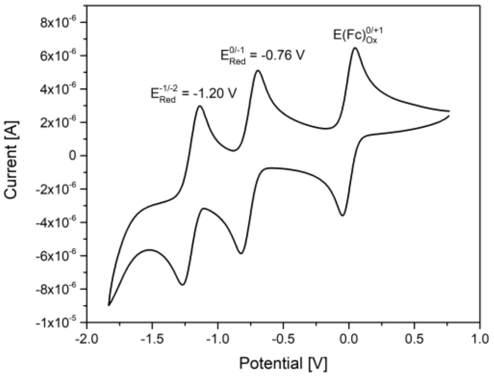

Figure S14. Cyclic voltammogram of **6**.

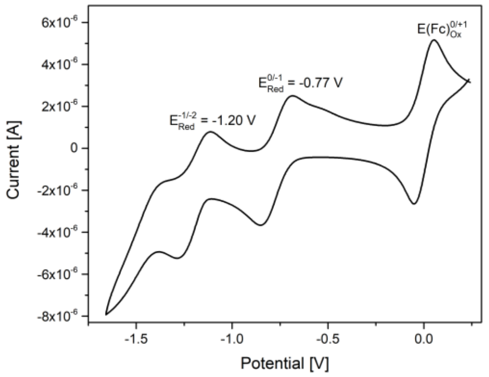

Figure S15. Cyclic voltammogram of **8**.

Calculations

	2	3a	3b	6	8
**LUMO**	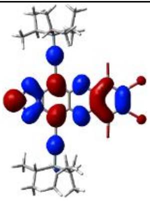<br>-4.19 eV	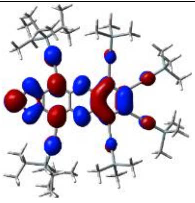<br>-3.94 eV	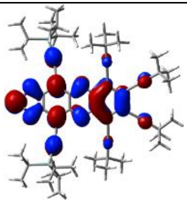<br>-3.75 eV	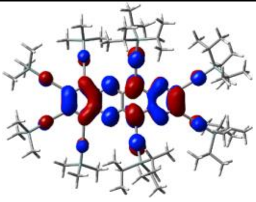<br>-3.72 eV	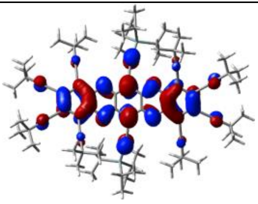<br>-3.68 eV
**HOMO**	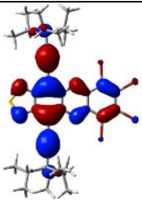<br>-5.97 eV	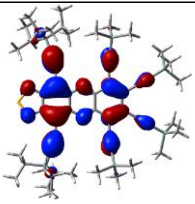<br>-5.65 eV	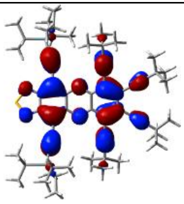<br>-5.45 eV	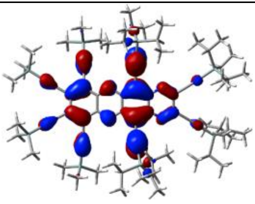<br>-5.57 eV	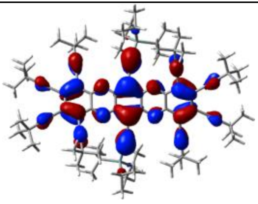<br>-5.21 eV
**Gap**	1.78 eV	1.71 eV	1.70 eV	1.85 eV	1.53 eV

Figure S16. Distribution of FMOs in **2**, **3a**, **3b**, **6** and **8**, calculated using TURBOMOLE B3LYP/def2TZVP//Gaussian 09 B3LYP/6-311++G**.

NMR-Spectra

***6,7,8,9-Tetrabromo-4,11-bis((triisopropylsilyl)ethynyl)-[1,2,5]thiadiazolo[3,4-b]phenazine* 2**

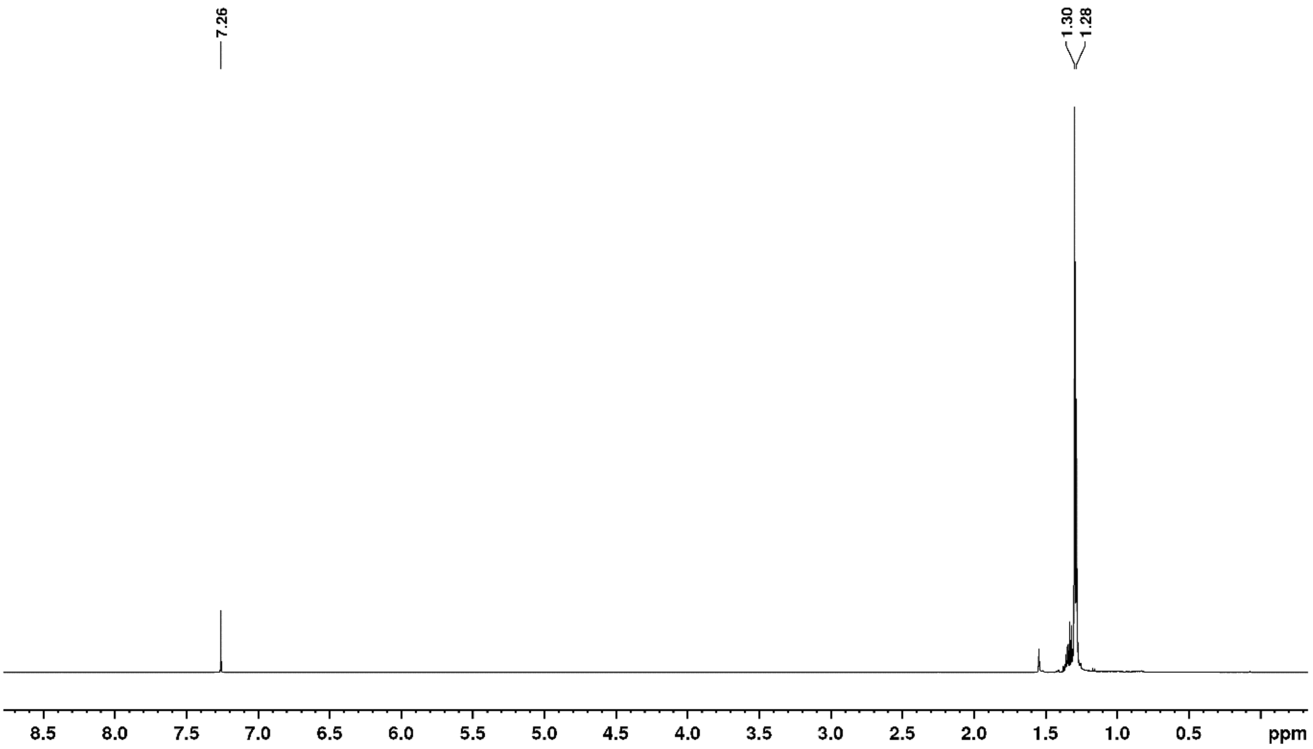

Figure S17. ^{1}H NMR (500 MHz, $CDCl_3$) of **2**.

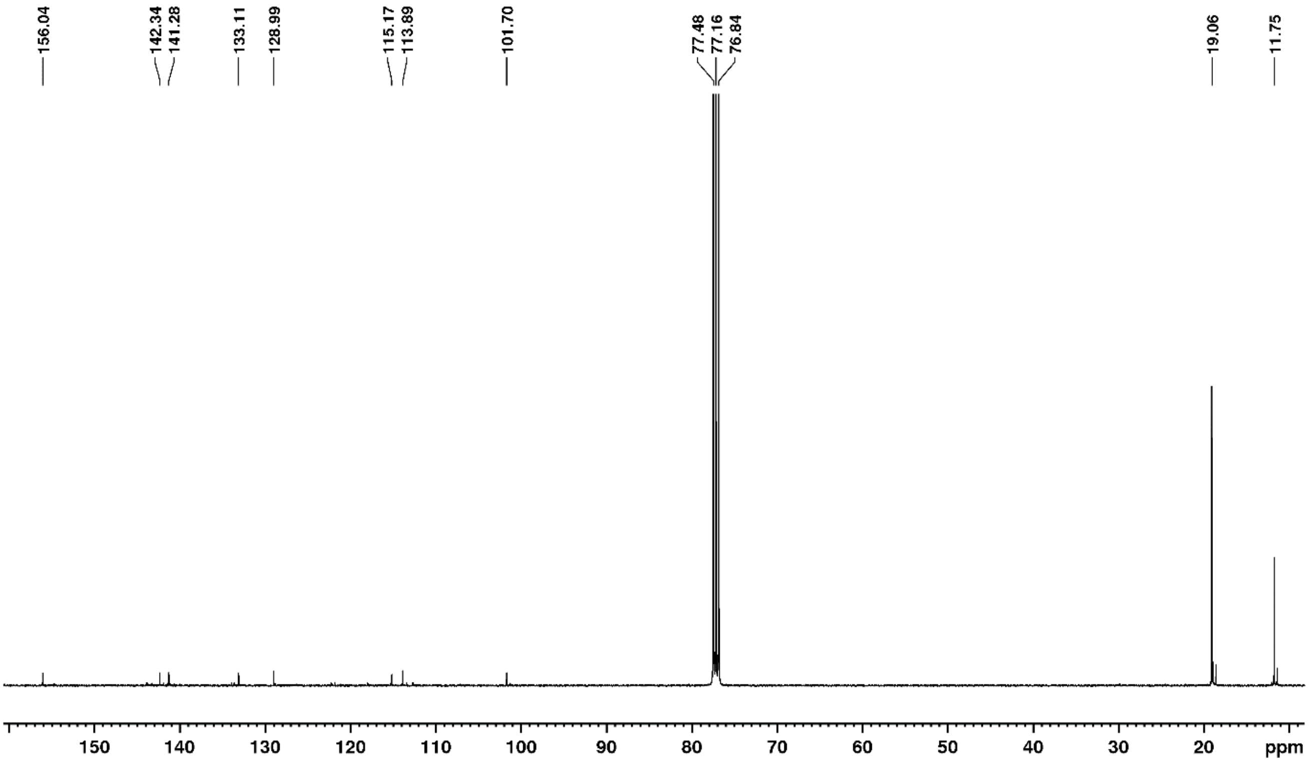

Figure S18. ^{13}C {^{1}H} NMR (100 MHz, $CDCl_3$) of **2**.

***4,11-bis((triisopropylsilyl)ethynyl)-6,7,8,9-tetrakis((trimethylsilyl)ethynyl)-5,10-dihydro-[1,2,5]thiadiazolo[3,4-b]phenazine* 3-H₂**

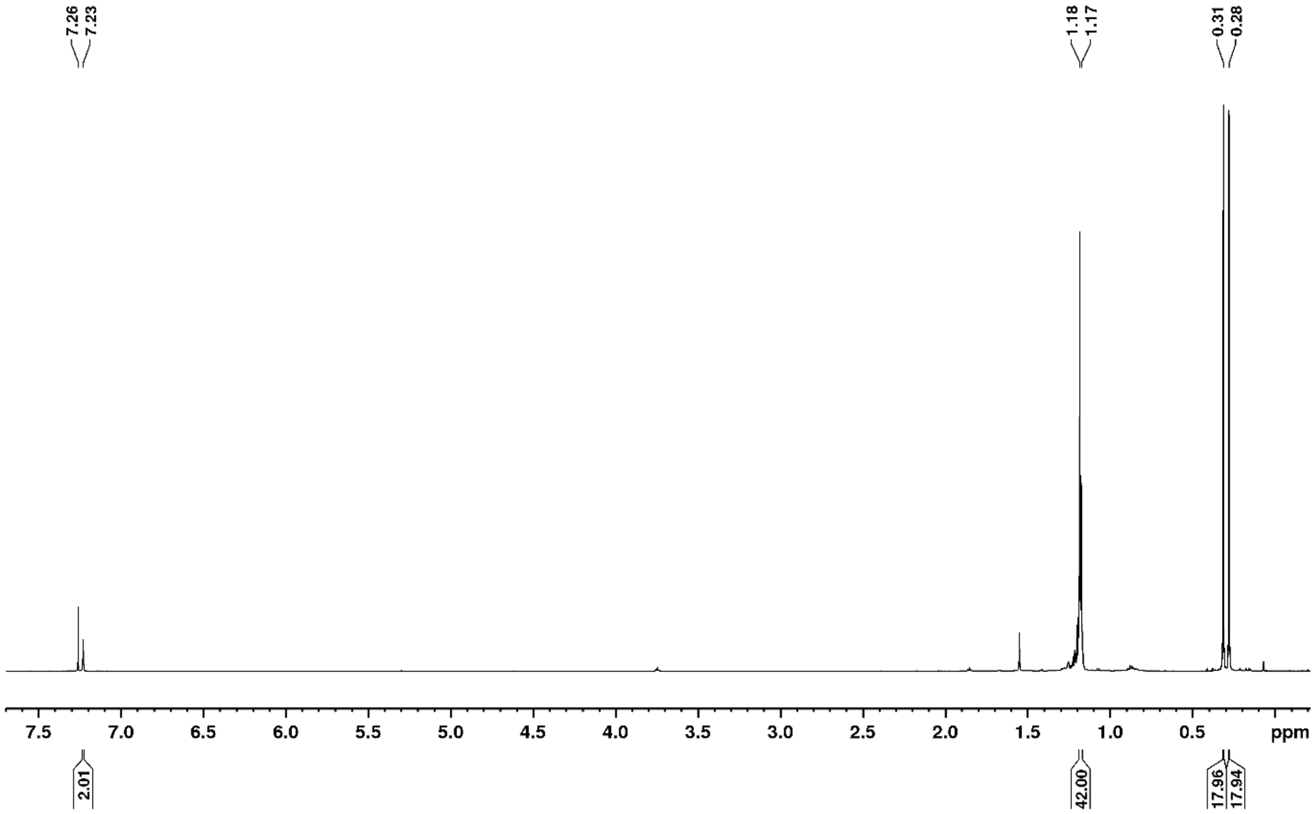

Figure S19. ^{1}H NMR (600 MHz, $CDCl_3$) of **3a-H$_2$**.

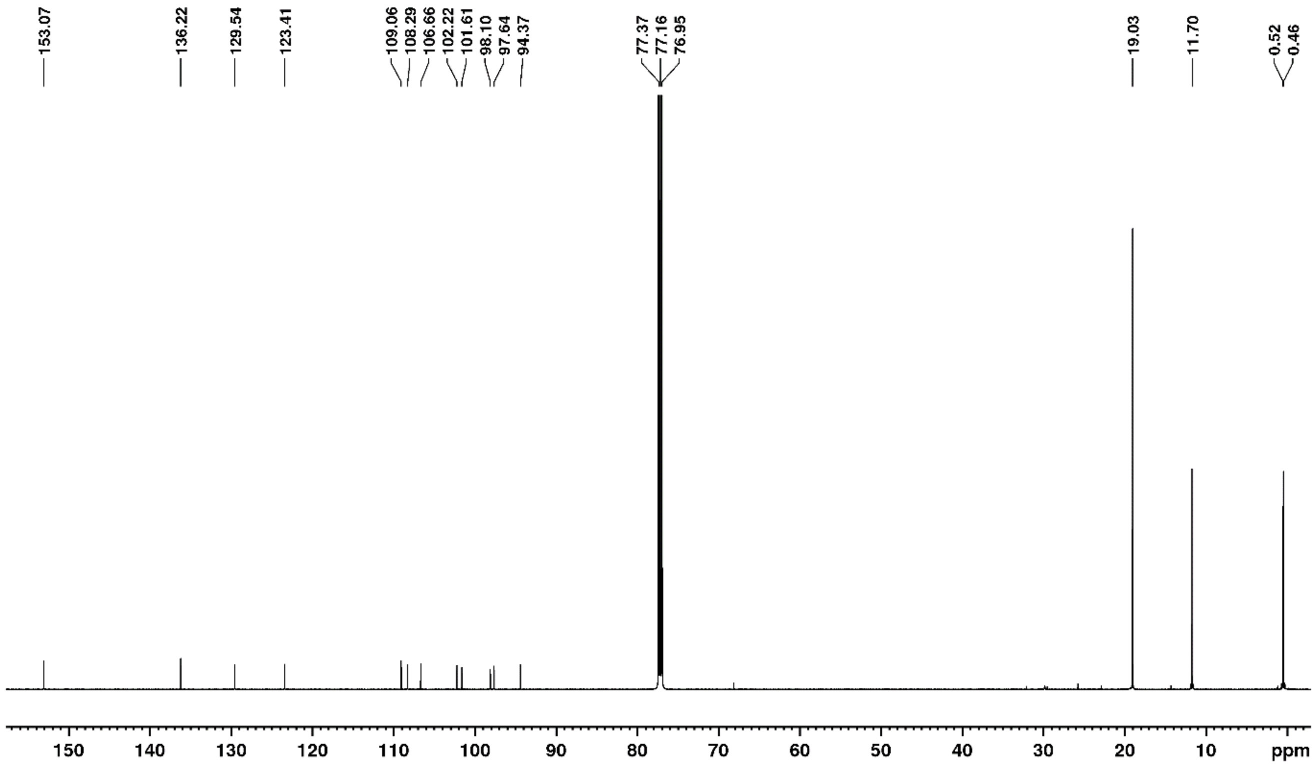

Figure S20. ^{13}C {^{1}H} NMR (150 MHz, $CDCl_3$) of **3a-H$_2$**.

***4,11-bis((triisopropylsilyl)ethynyl)-6,7,8,9-tetrakis((trimethylsilyl)ethynyl)-[1,2,5]thiadiazolo[3,4-b]phenazine* 3a**

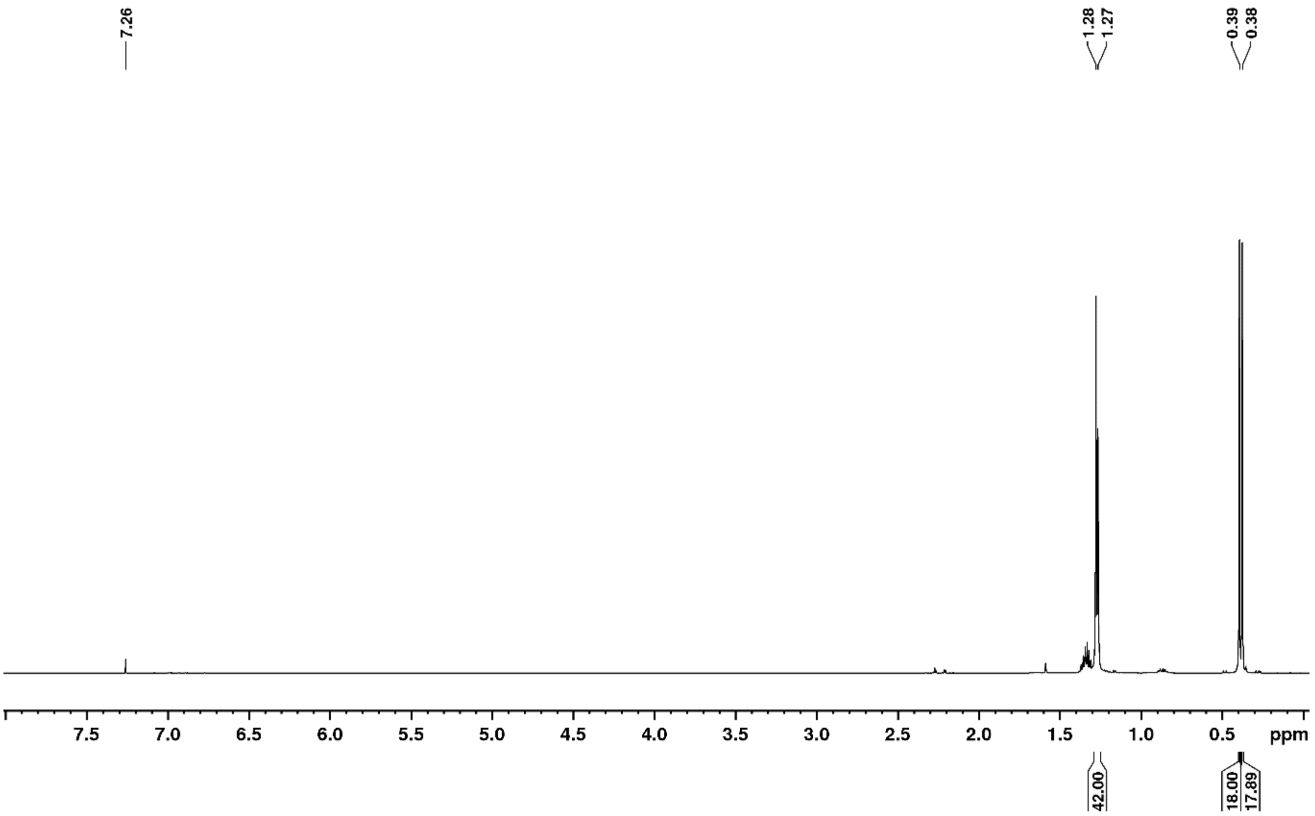

Figure S21. ^{1}H NMR (600 MHz, $CDCl_3$) of **3a**.

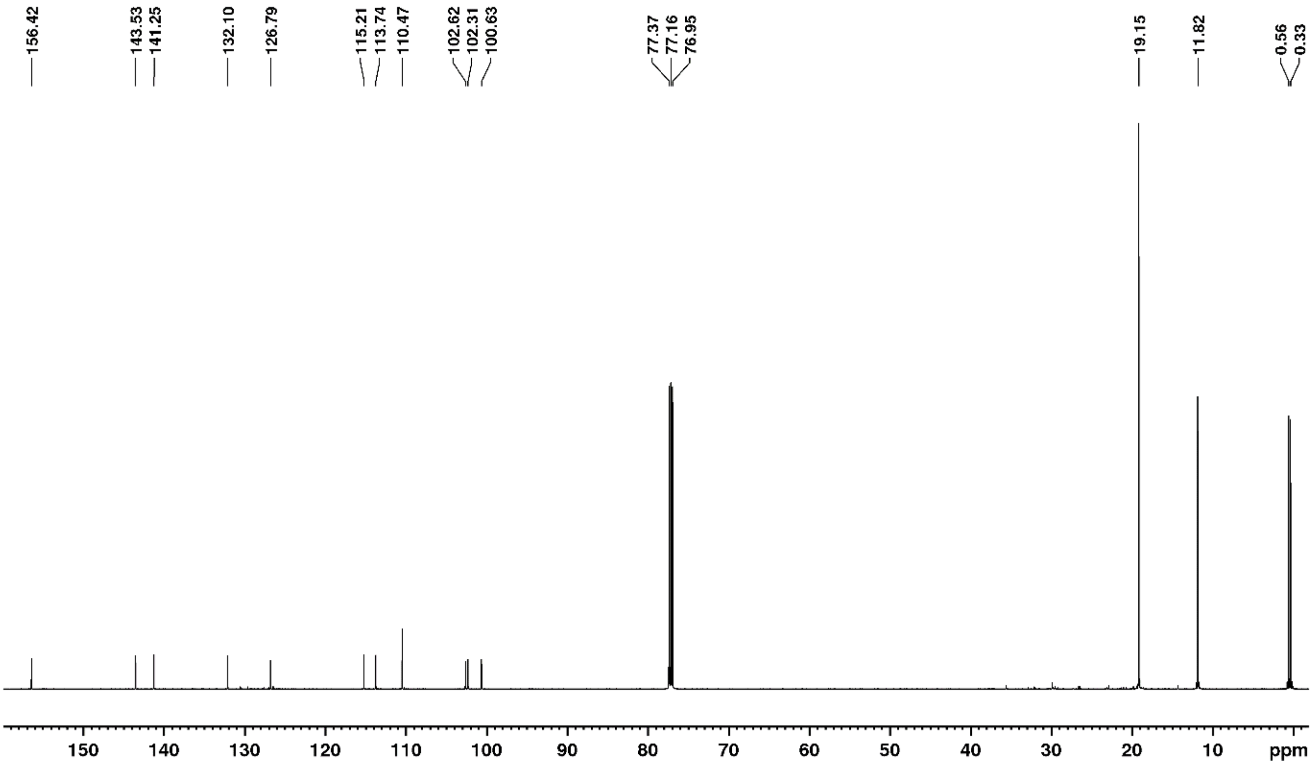

Figure S22. ^{13}C {^{1}H} NMR (150 MHz, $CDCl_3$) of **3a**.

6,7,8,9-tetrakis(3,3-dimethylbut-1-yn-1-yl)-4,11-bis((triisopropylsilyl)ethynyl)-5,10-dihydro-[1,2,5]thiadiazolo[3,4-b]phenazine

3b-H2

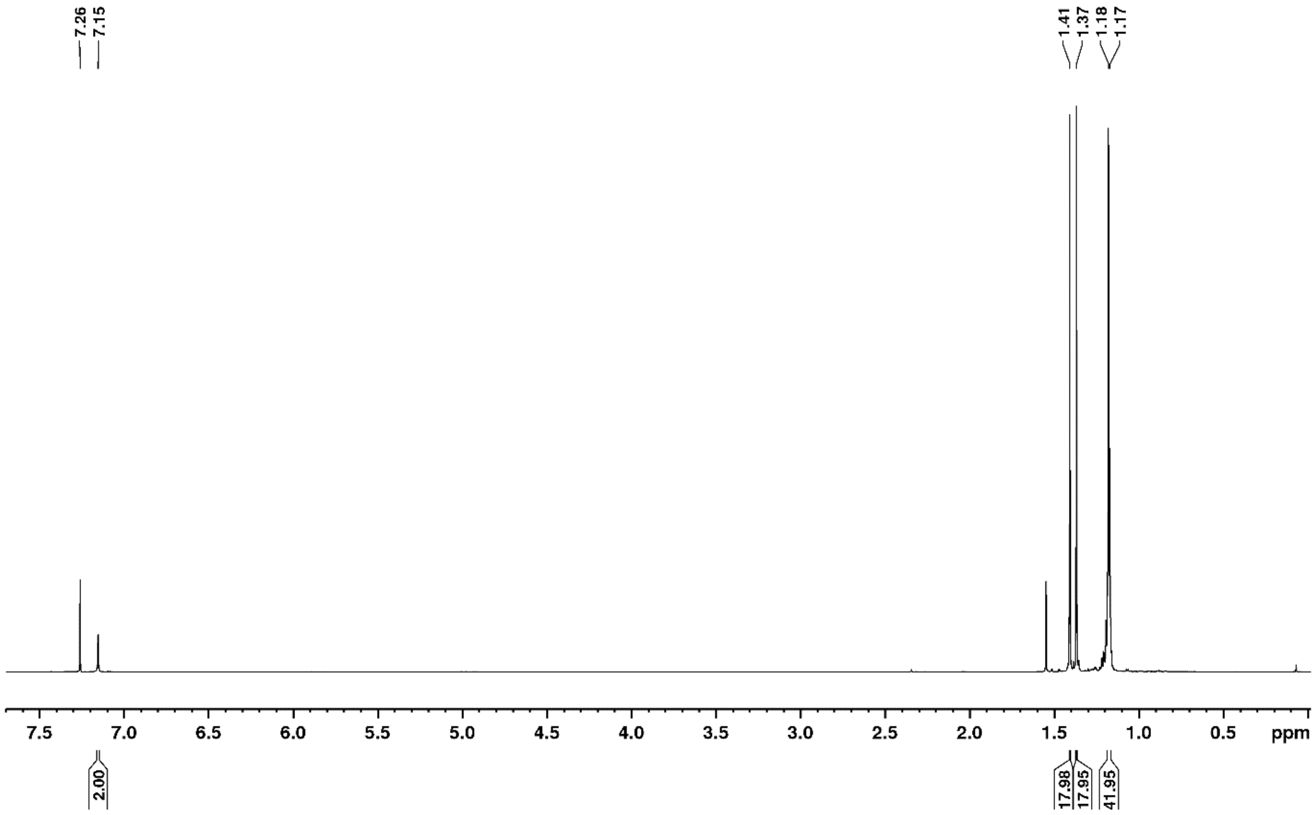

Figure S23. ^{1}H NMR (600 MHz, $CDCl_3$) of **3b-H2**.

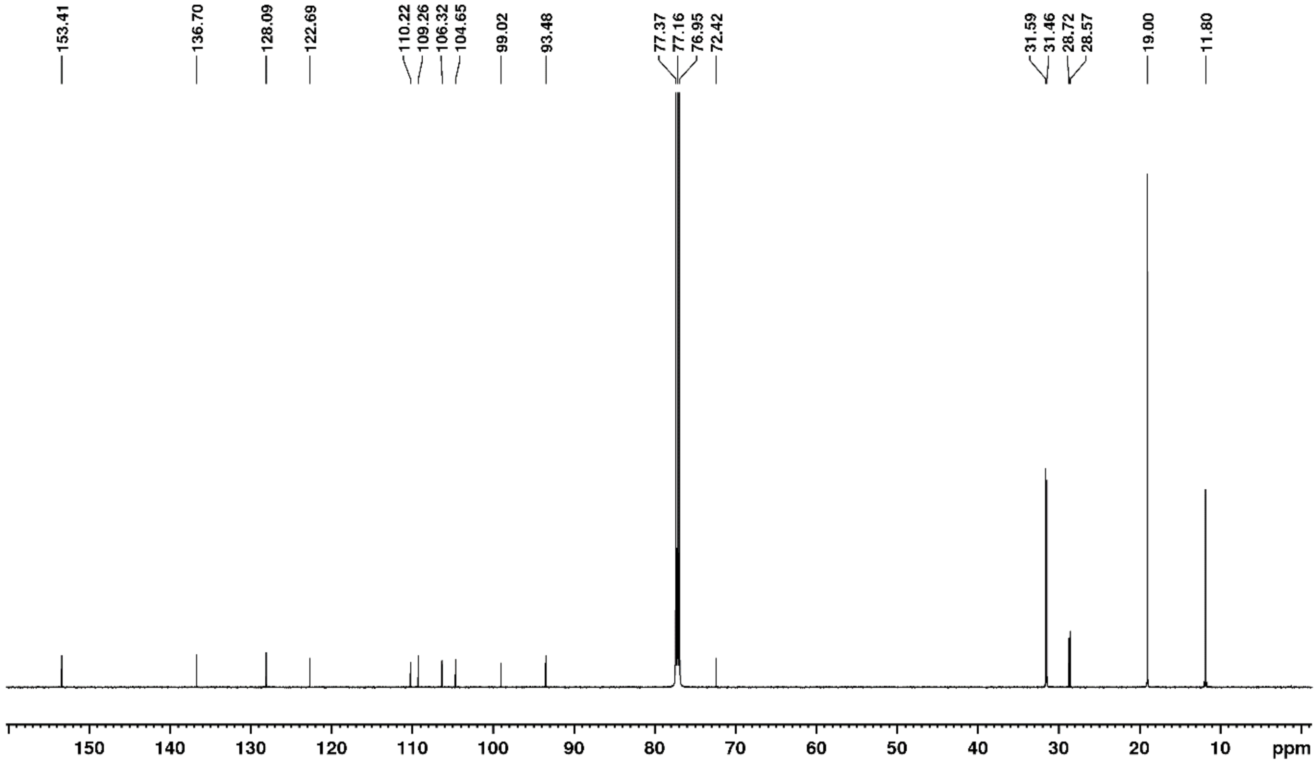

Figure S24. ^{13}C {^{1}H} NMR (150 MHz, $CDCl_3$) of **3b-H2**.

***6,7,8,9-tetrakis(3,3-dimethylbut-1-yn-1-yl)-4,11-bis((triisopropylsilyl)ethynyl)-[1,2,5]thiadiazolo[3,4-b]phenazine* 3b**

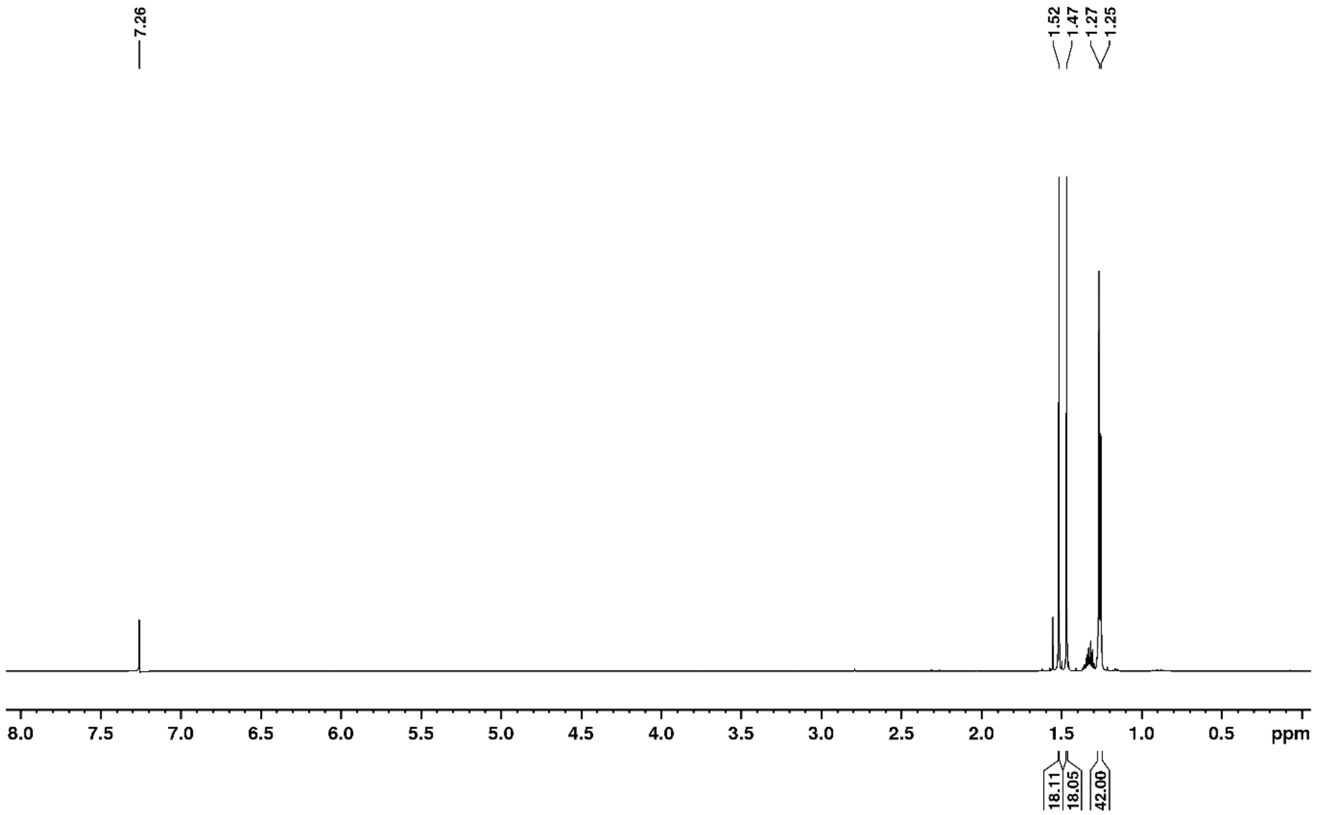

Figure S25. ^{1}H NMR (600 MHz, $CDCl_3$) of **3b**.

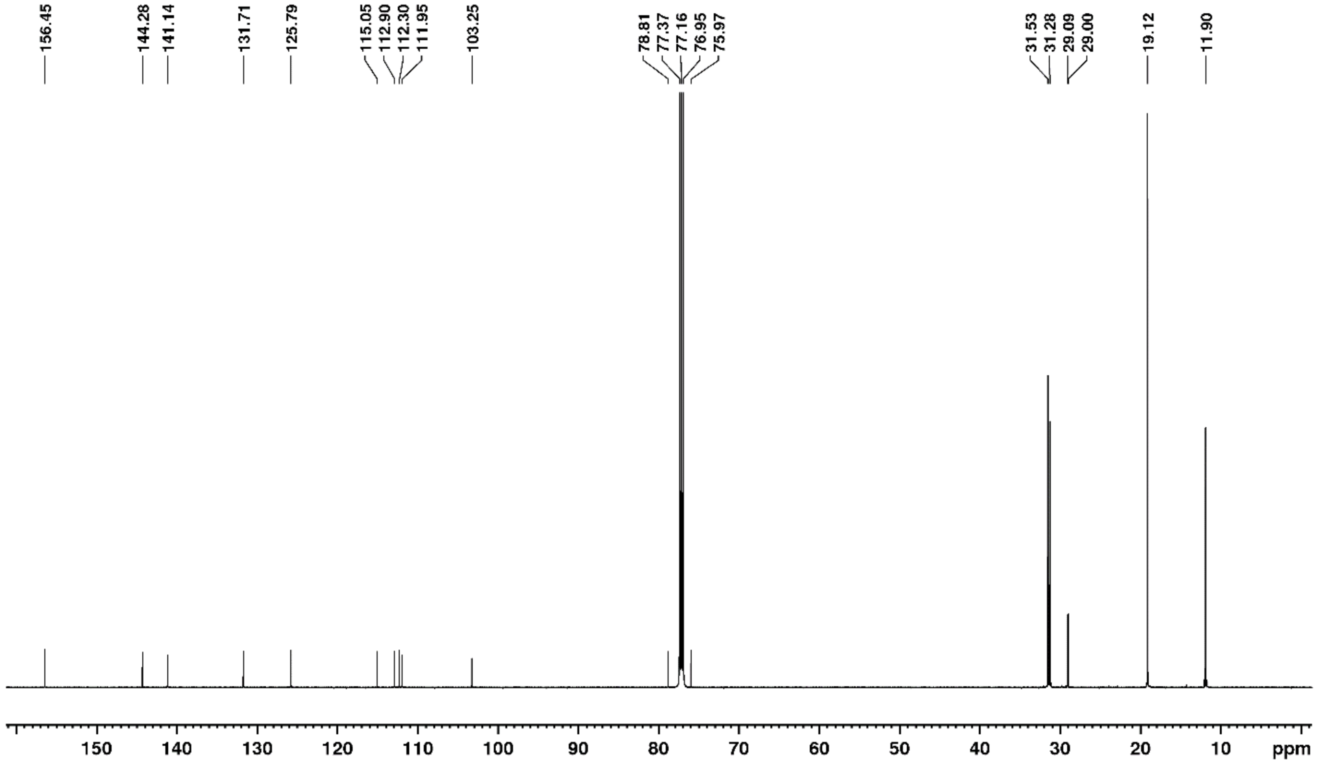

Figure S26. ^{13}C {^{1}H} NMR (150 MHz, $CDCl_3$) of **3b**.

***1,4-bis((triisopropylsilyl)ethynyl)-6,7,8,9-tetrakis((trimethylsilyl)ethynyl)phenazine-2,3-diamine* 4a**

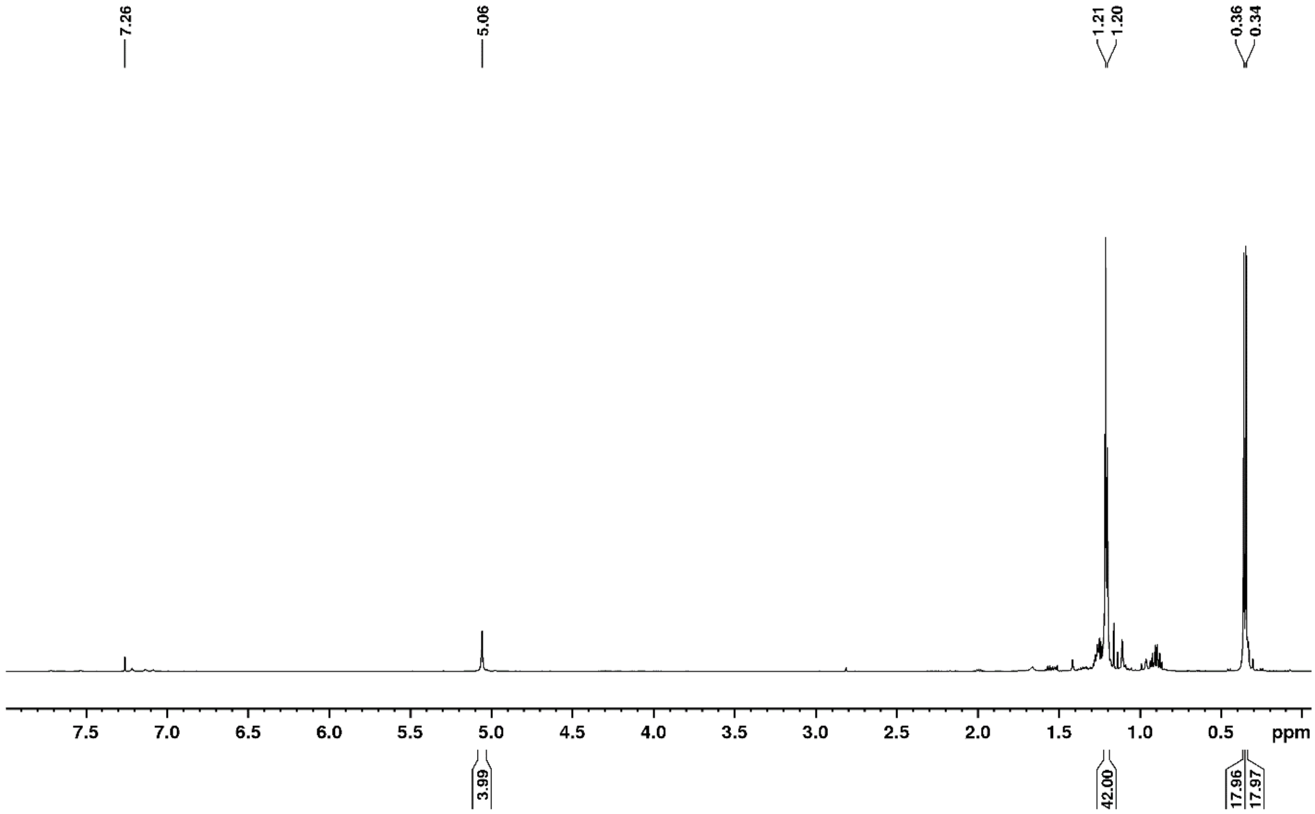

Figure S27. ^{1}H NMR (600 MHz, $CDCl_3$) of **4a**.

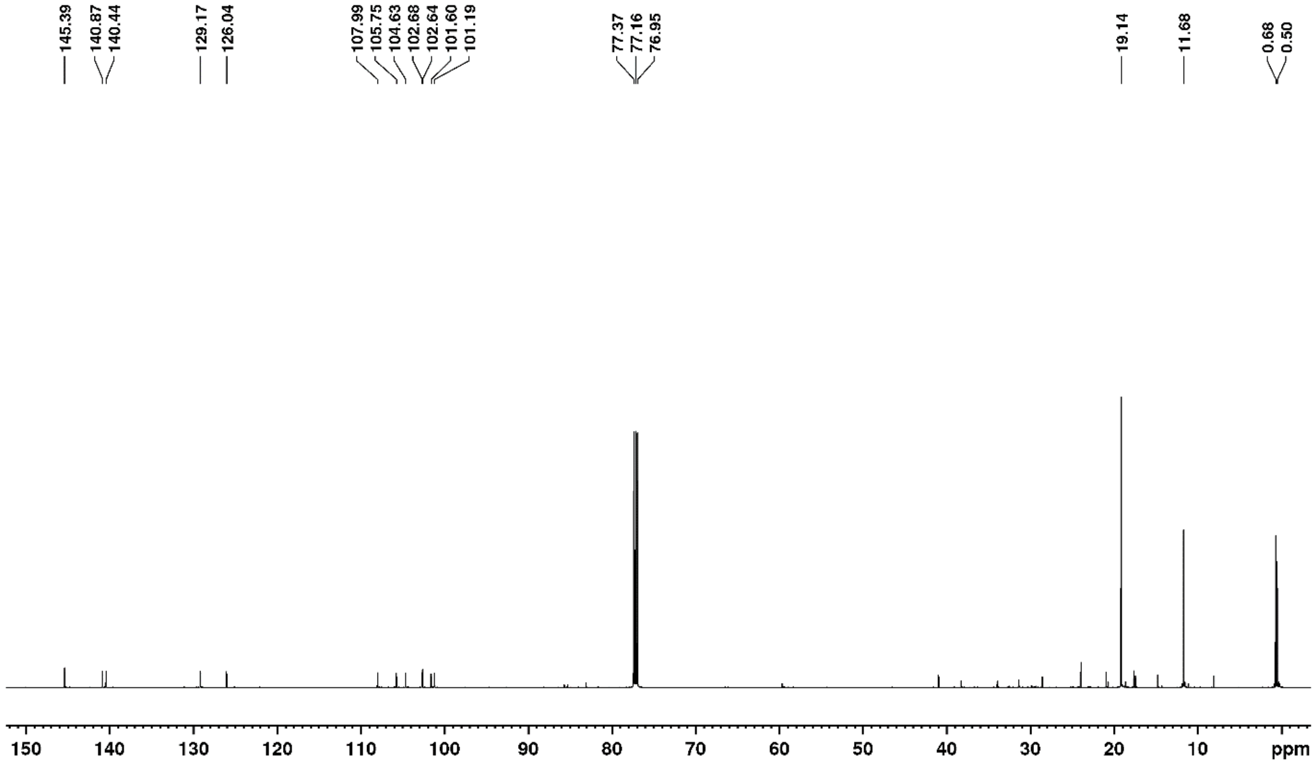

Figure S28. ^{13}C {^{1}H} NMR (150 MHz, $CDCl_3$) of **4a**.

***6,7,8,9-tetrakis(3,3-dimethylbut-1-yn-1-yl)-1,4-bis((triisopropylsilyl)ethynyl)phenazine-2,3-diamine* 4b**

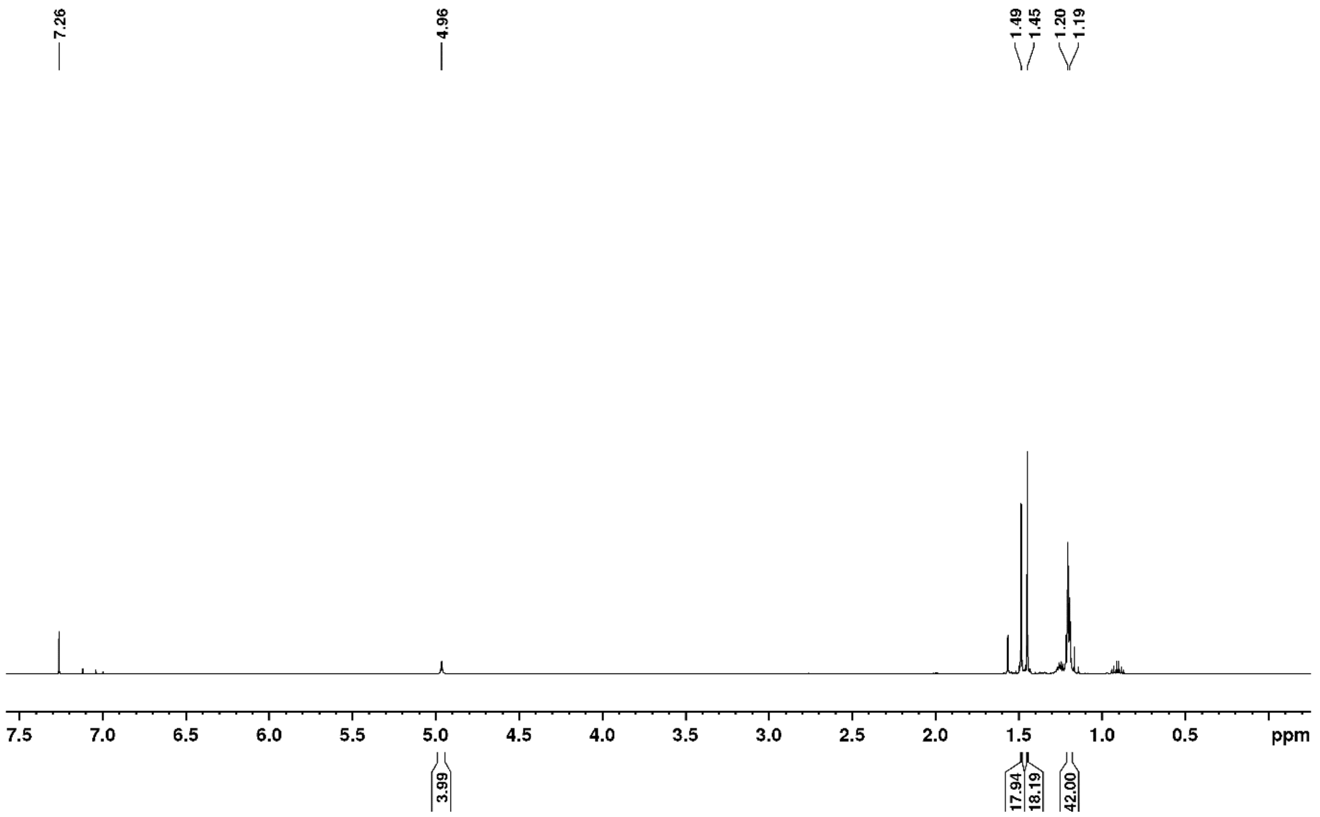

Figure S29. ^{1}H NMR (600 MHz, $CDCl_3$) of **4b**.

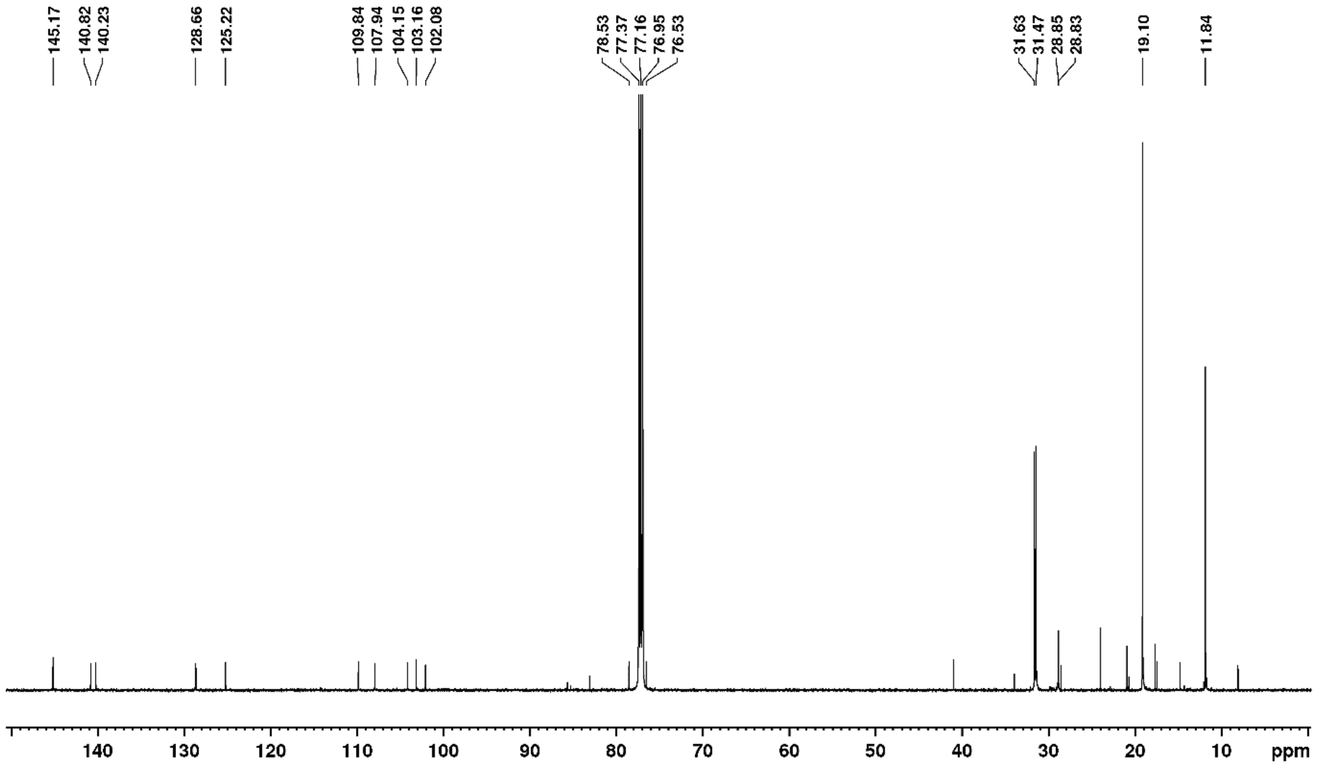

Figure S30. ^{13}C {^{1}H} NMR 150 MHz, $CDCl_3$) of **4b**.

***2,3,5,12-tetrakis((triisopropylsilyl)ethynyl)-7,8,9,10-tetrakis((trimethylsilyl)ethynyl)-1,2-dihydropyrazino[2,3-b]phenazine* 6**

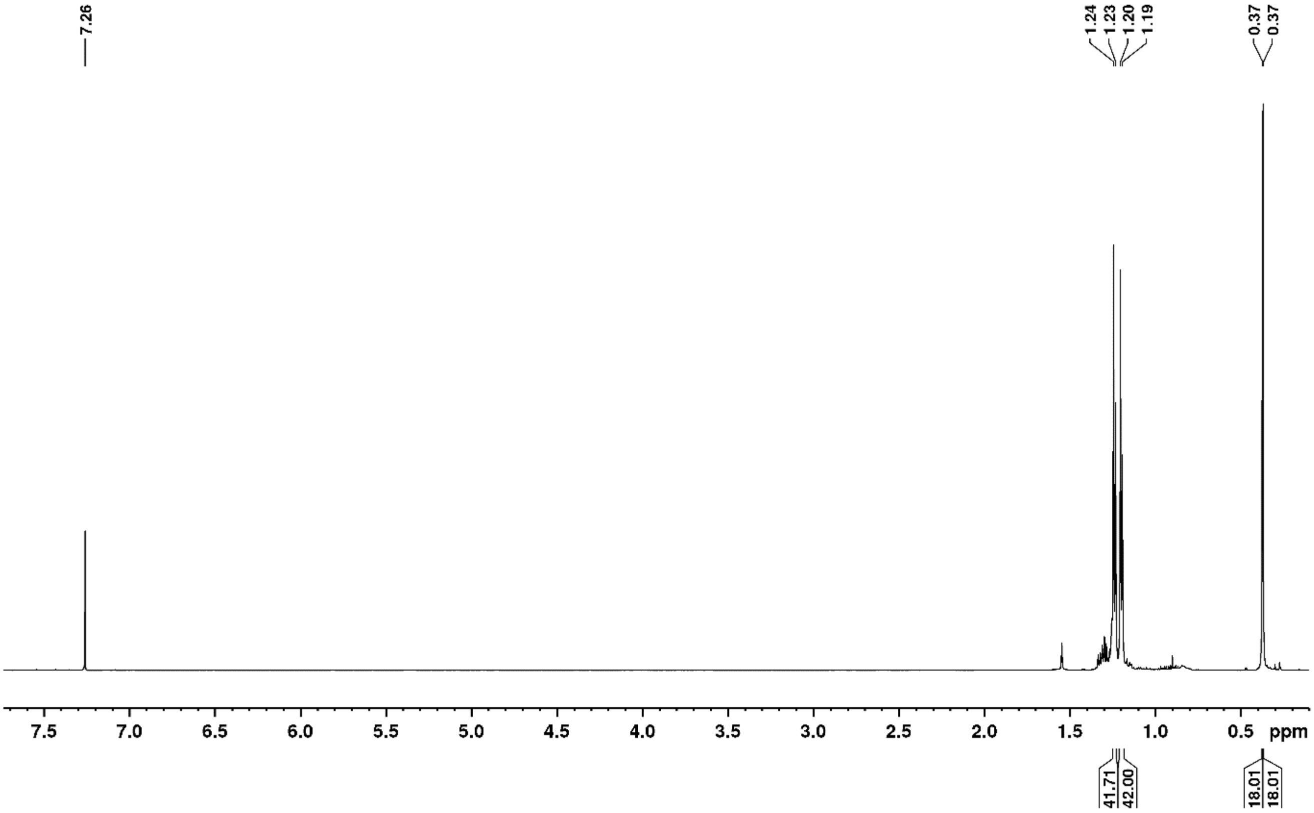

Figure S31. ^{1}H NMR (600 MHz, $CDCl_3$) of **6**.

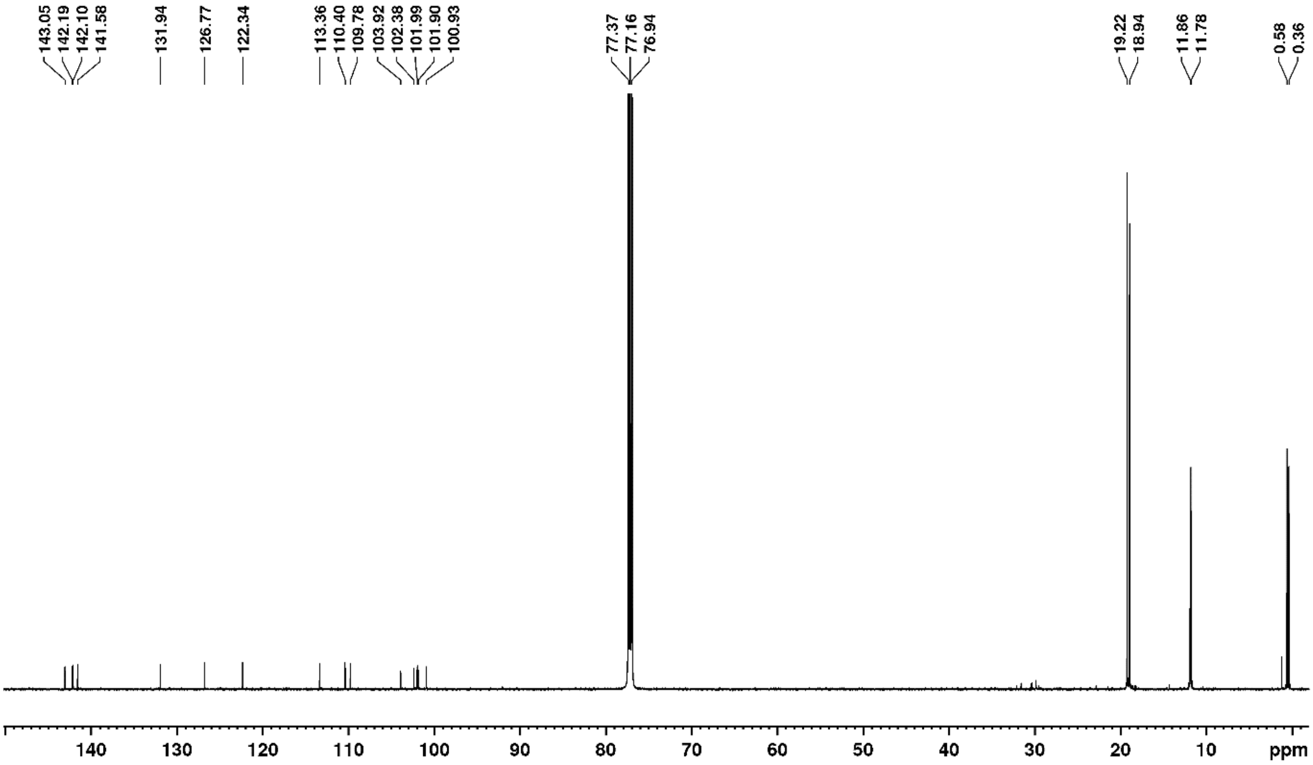

Figure S32. ^{13}C {^{1}H} NMR (150 MHz, $CDCl_3$) of **6**.

***1,2,3,4-tetrabromo-6,13-bis((triisopropylsilyl)ethynyl)-8,9,10,11-tetrakis((trimethylsilyl)ethynyl)-quinoxalino[2,3-b]phenazine* 7a**

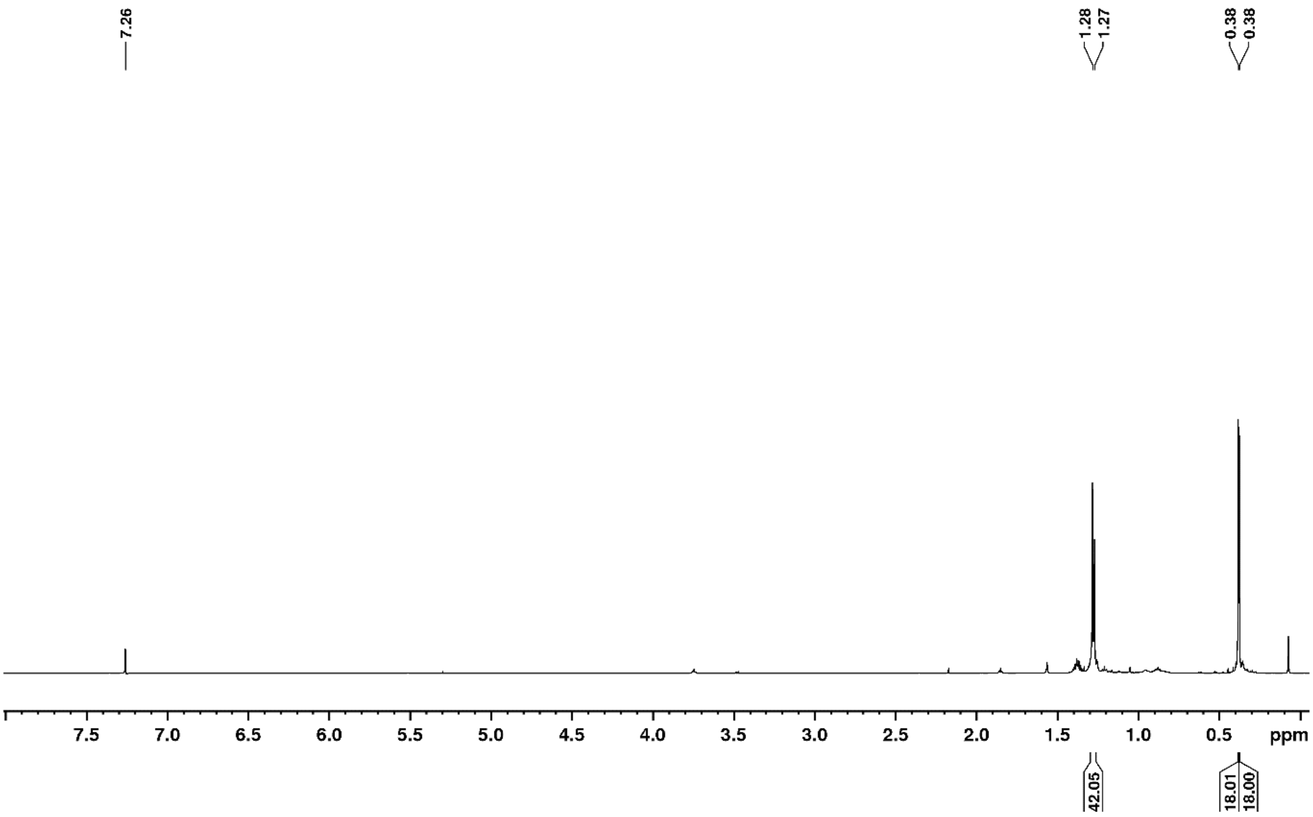

Figure S33. ^{1}H NMR (600 MHz, $CDCl_3$) of **7a**.

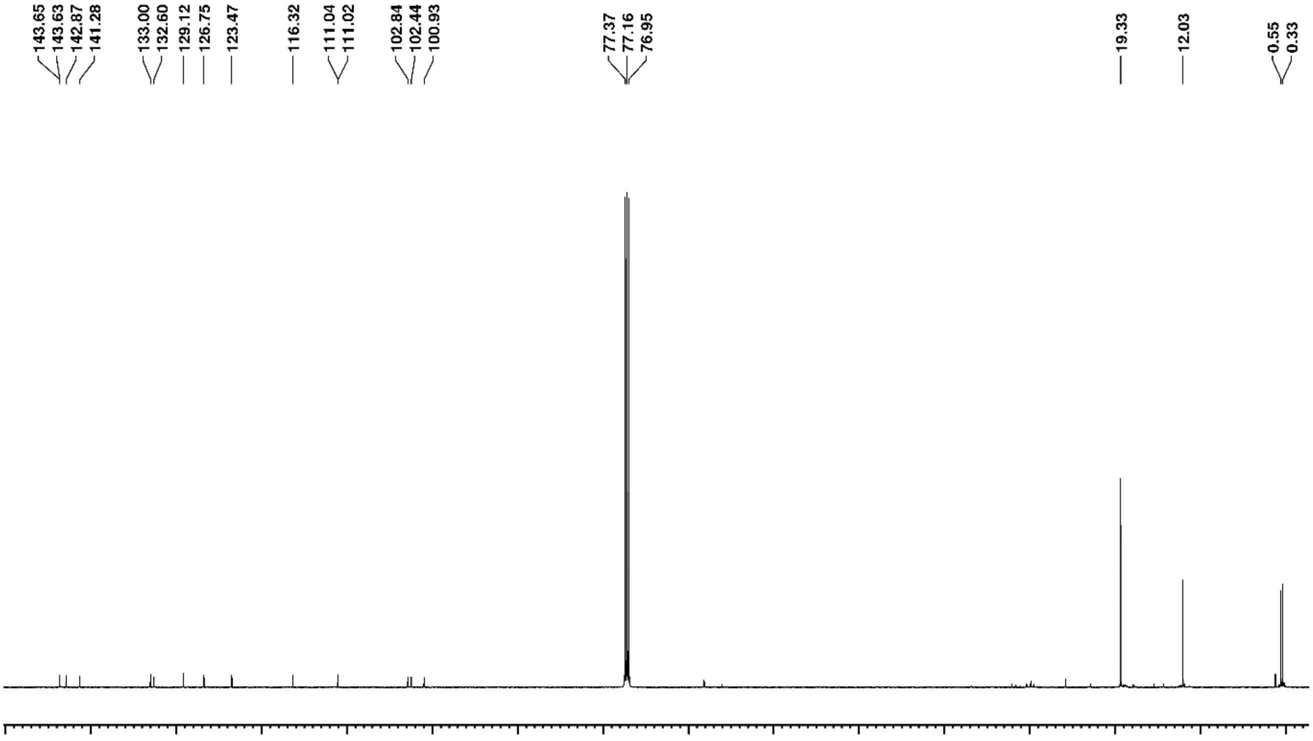

Figure S34. ^{13}C {^{1}H} NMR (150 MHz, $CDCl_3$) of **7a**.

***1,2,3,4-tetrabromo-8,9,10,11-tetrakis(3,3-dimethylbut-1-yn-1-yl)-6,13-bis((triisopropylsilyl)ethynyl)-5,14-dihydroquinoxalino-[2,3-b]phenazine* 7b-H₂**

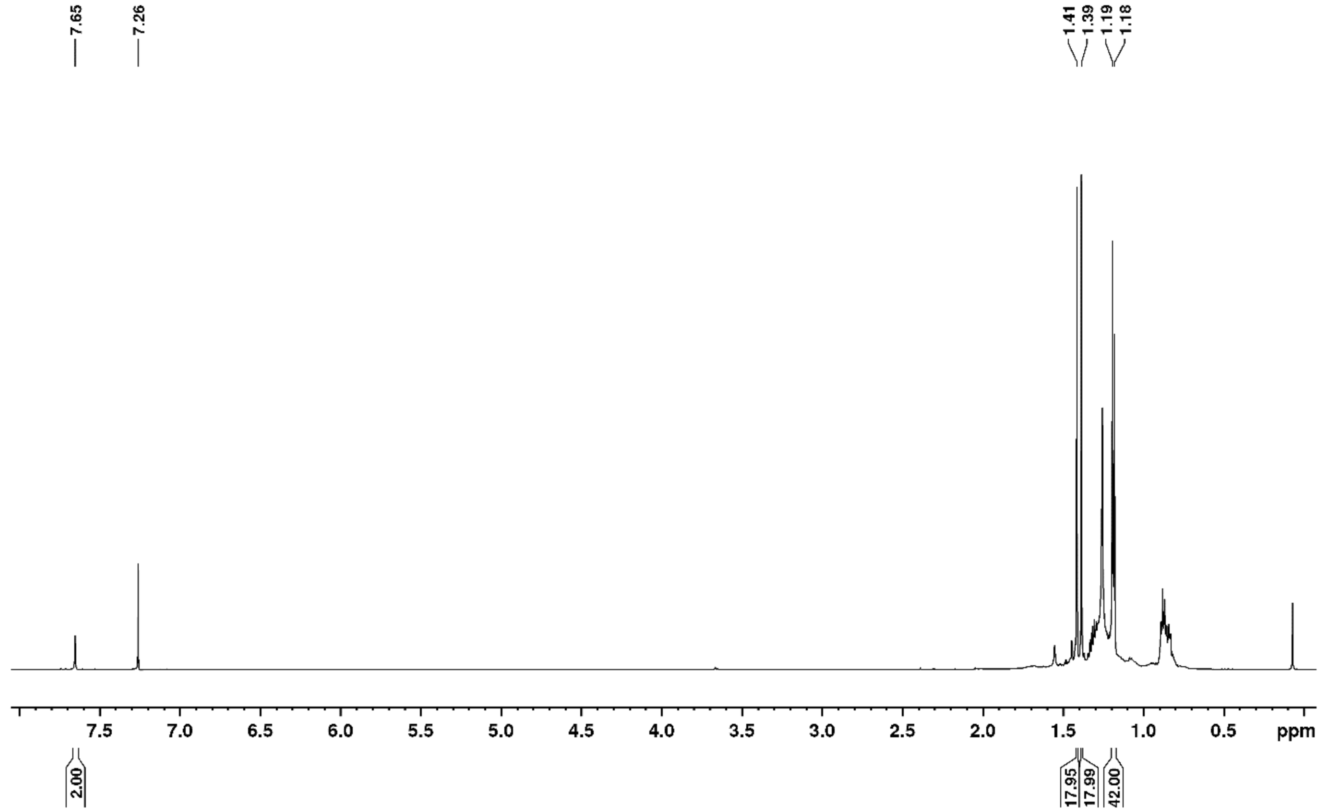

Figure S35. ^{1}H NMR (600 MHz, $CDCl_3$) of **7b-H$_2$**.

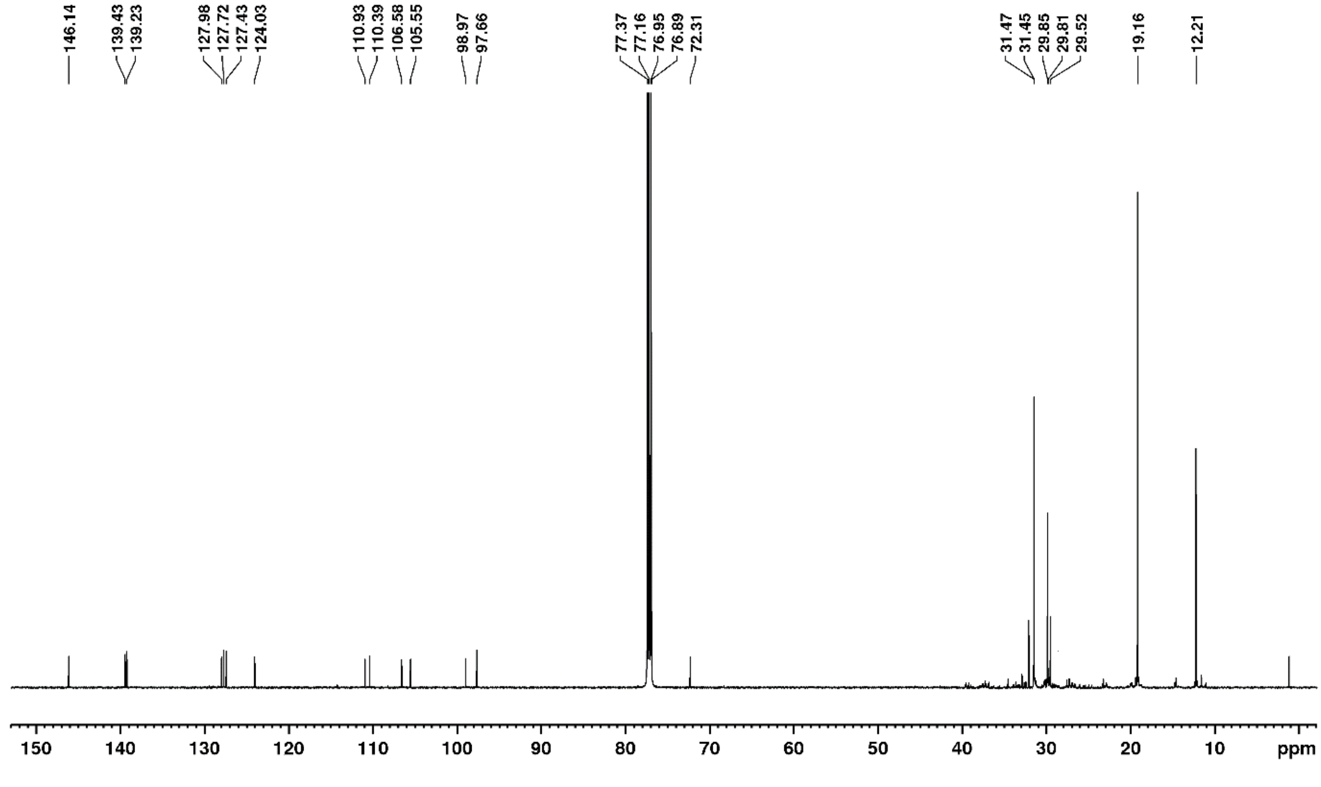

Figure S36. ^{13}C {^{1}H} NMR (150 MHz, $CDCl_3$) of **7b-H$_2$**.

***1,2,3,4,8,9,10,11-octakis(3,3-dimethylbut-1-yn-1-yl)-6,13-bis((triisopropylsilyl)ethynyl)-5,14-dihydroquinoxalino[2,3-b]phenazine* 8-H$_2$**

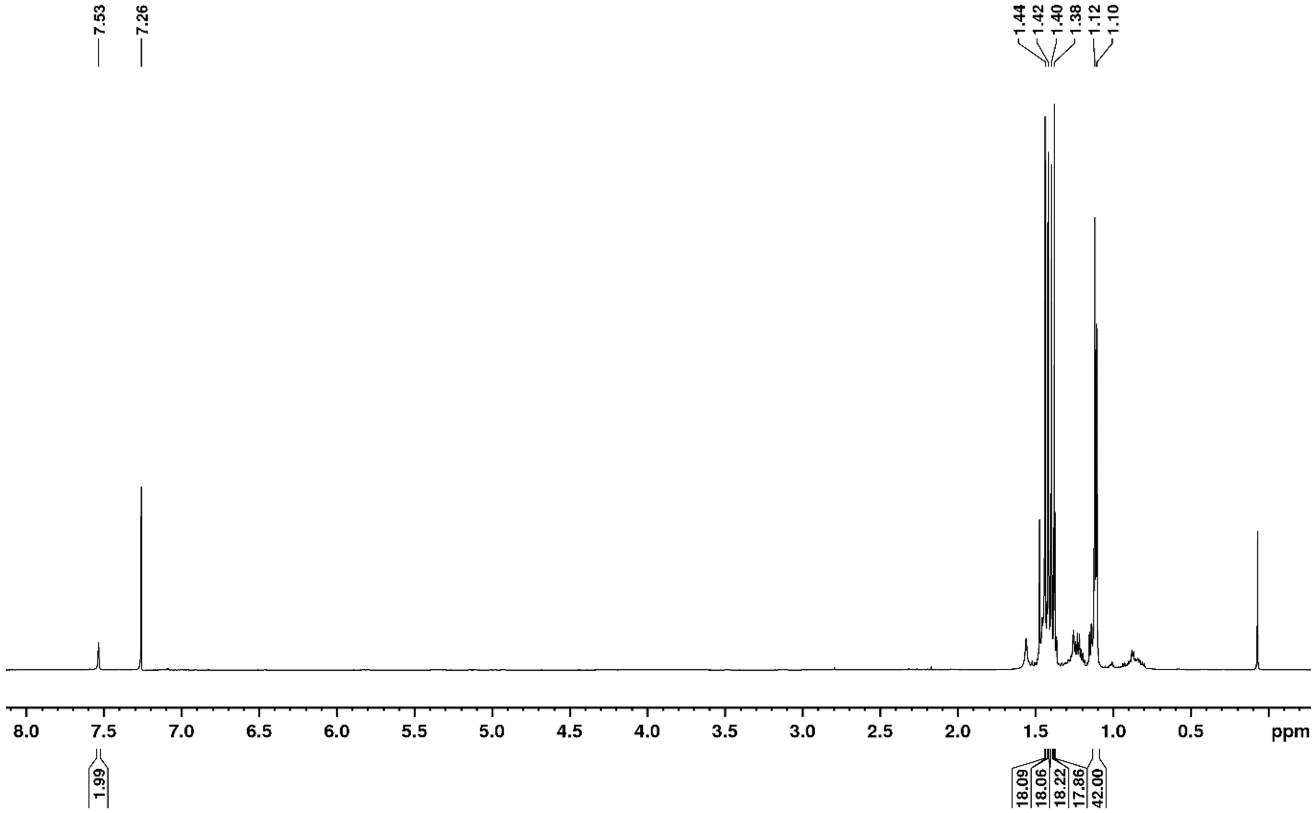

Figure S37. ^{1}H NMR (600 MHz, $CDCl_3$) of **8-H$_2$**.

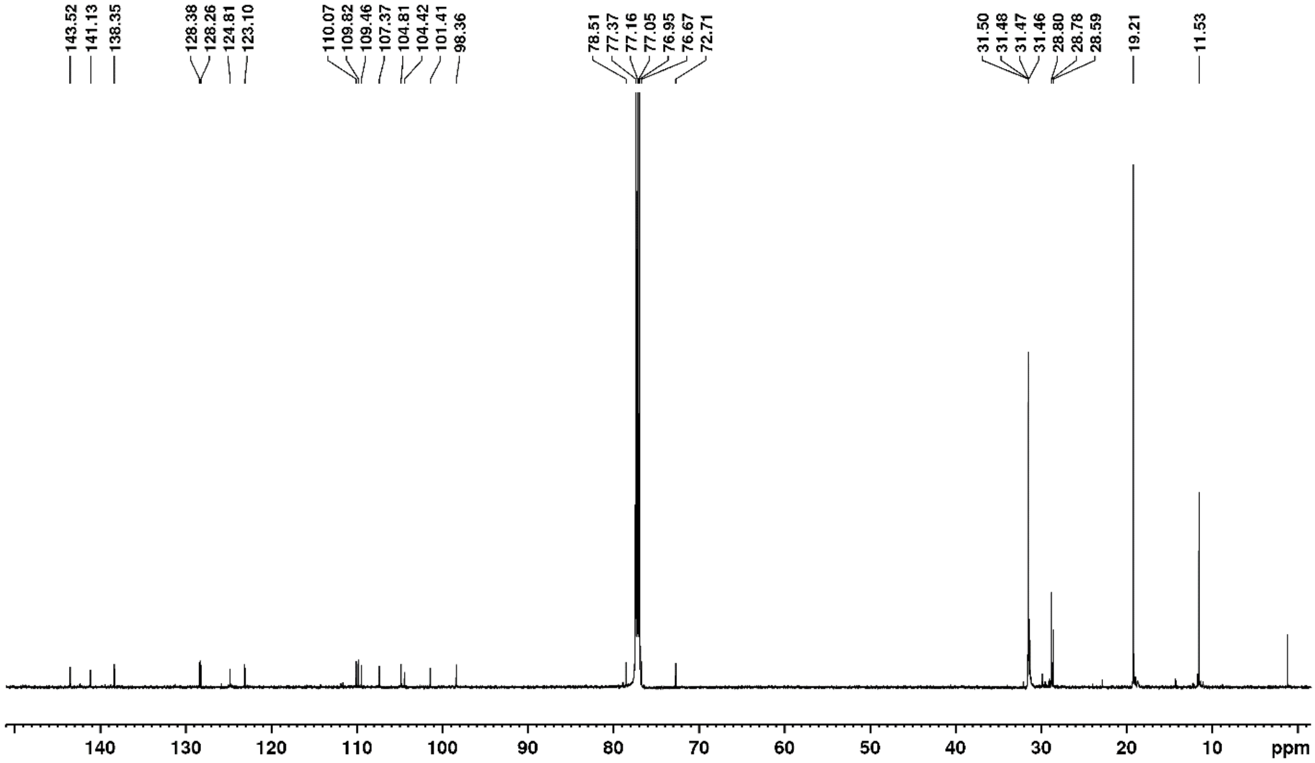

Figure S38. ^{13}C {^{1}H} NMR (150 MHz, $CDCl_3$) of **8-H$_2$**.

***1,2,3,4,8,9,10,11-octakis(3,3-dimethylbut-1-yn-1-yl)-6,13-bis((triisopropylsilyl)ethynyl)-quinoxalino[2,3-b]phenazine* 8**

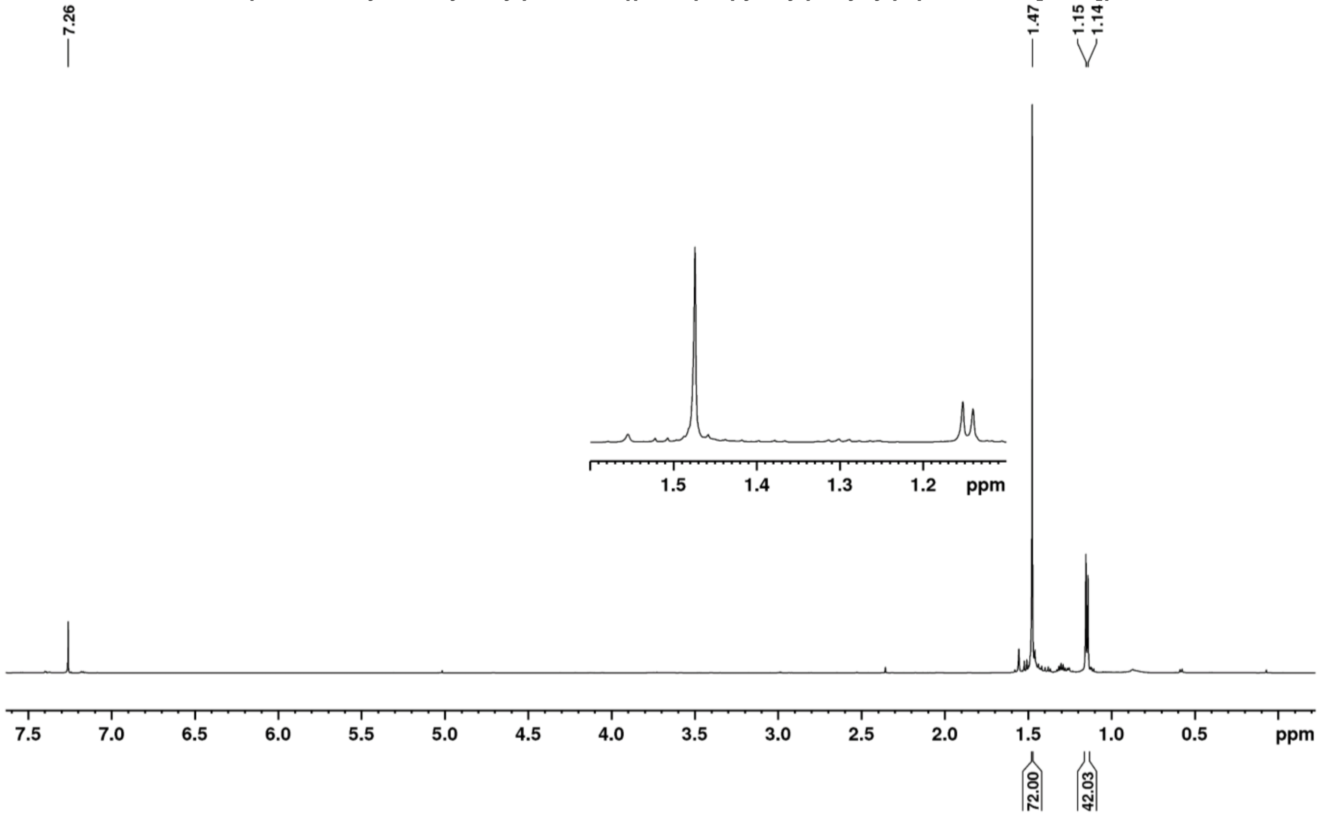

Figure S39. ^{1}H NMR (600 MHz, $CDCl_3$) of **8**.

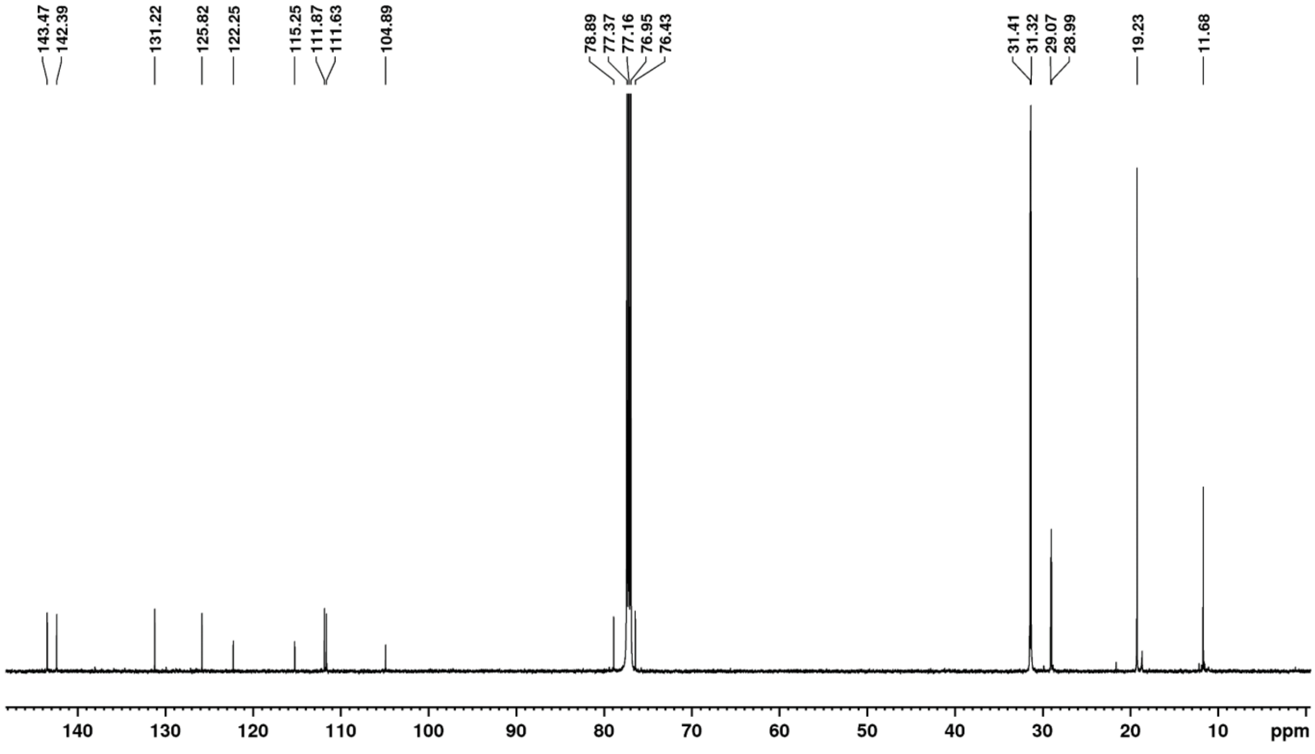

Figure S40. $^{13}C\{^{1}H\}$ NMR (150 MHz, $CDCl_3$) of **8**.

IR-Spectra

***6,7,8,9-Tetrabromo-4,11-bis((triisopropylsilyl)ethynyl)-[1,2,5]thiadiazolo[3,4-b]phenazine* 2**

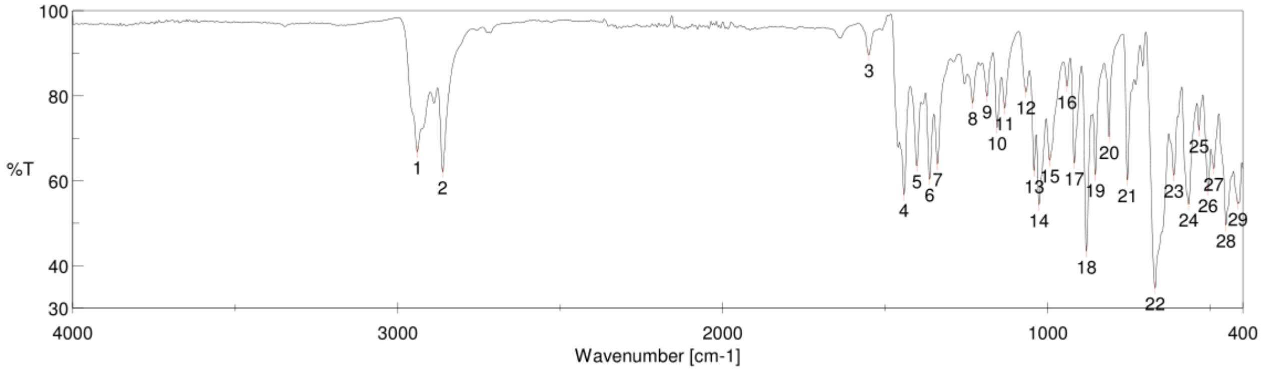

[Result of Peak Picking]

No.	Position	Intensity	No.	Position	Intensity	No.	Position	Intensity
1	2939.95	66.7594	2	2861.84	62.004	3	1550.49	89.5623
4	1442.49	56.6389	5	1402.96	63.4511	6	1363.43	60.2929
7	1339.32	63.9017	8	1231.33	78.2948	9	1186.97	79.8746
10	1156.12	72.4317	11	1132.97	77.022	12	1067.41	80.9025
13	1042.34	62.2957	14	1026.91	54.2652	15	994.125	64.7536
16	941.092	82.1917	17	918.914	64.0789	18	881.309	43.2936
19	854.311	61.3706	20	811.885	70.3189	21	754.995	60.0841
22	670.142	34.6419	23	612.288	61.2556	24	566.969	54.391
25	535.15	71.7427	26	507.187	57.764	27	489.831	62.8046
28	452.225	49.4567	29	415.585	54.561			

Figure S41. IR-Spectrum of **2**.

***4,11-bis((triisopropylsilyl)ethynyl)-6,7,8,9-tetrakis((trimethylsilyl)ethynyl)-5,10-dihydro-[1,2,5]thiadiazolo[3,4-b]phenazine* 3a-H_2**

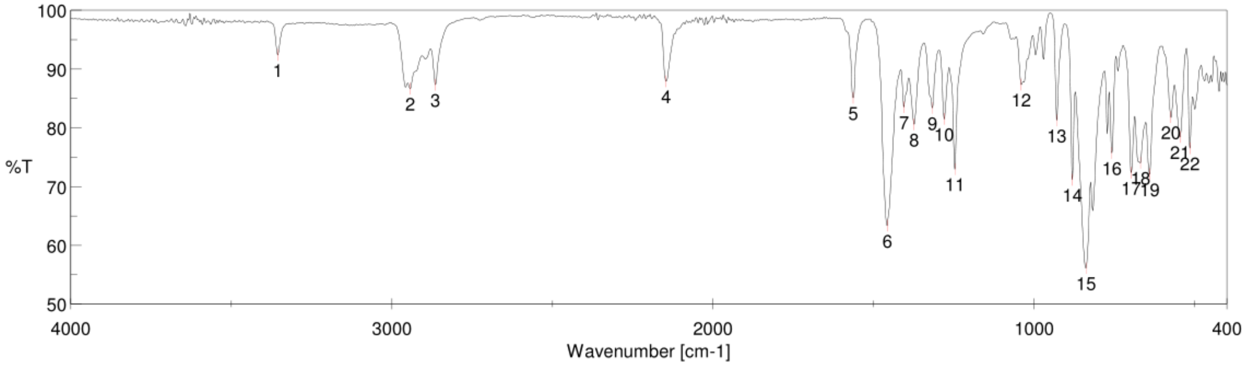

[Result of Peak Picking]

No.	Position	Intensity	No.	Position	Intensity	No.	Position	Intensity
1	3355.53	92.3756	2	2942.84	86.6525	3	2864.74	87.3164
4	2146.38	87.8914	5	1563.99	85.0935	6	1457.92	63.3392
7	1405.85	83.5026	8	1374.03	80.5737	9	1317.14	83.3299
10	1280.5	81.4957	11	1246.75	72.9347	12	1040.41	87.3321
13	929.521	81.2483	14	881.309	71.1366	15	838.883	56.1073
16	757.888	75.6928	17	698.105	72.3221	18	669.178	74.0124
19	640.251	72.1054	20	574.683	81.7918	21	545.756	78.4297
22	515.865	76.4948						

Figure S42. IR-Spectrum of **3a-H_2**.

***4,11-bis((triisopropylsilyl)ethynyl)-6,7,8,9-tetrakis((trimethylsilyl)ethynyl)-[1,2,5]thiadiazolo[3,4-b]phenazine* 3a**

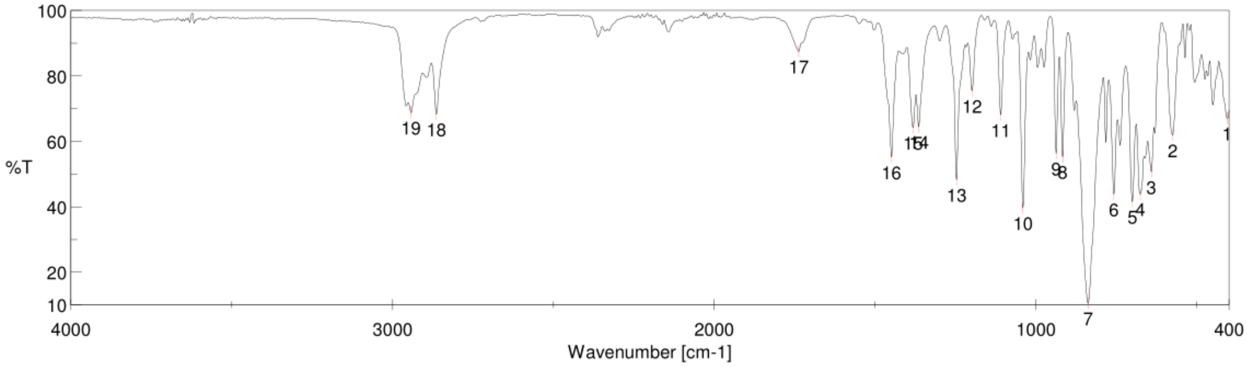

[Result of Peak Picking]

No.	Position	Intensity	No.	Position	Intensity	No.	Position	Intensity
1	405.942	67.0224	2	575.647	61.8316	3	641.215	50.5976
4	675.928	43.8654	5	700.998	41.4959	6	757.888	43.808
7	837.919	10.3845	8	916.986	55.2279	9	937.235	56.2307
10	1040.41	39.5949	11	1109.83	68.0403	12	1198.54	75.3886
13	1246.75	48.2417	14	1364.39	64.3966	15	1381.75	64.131
16	1449.24	55.0951	17	1737.55	87.4954	18	2863.77	68.2489
19	2942.84	68.7437						

Figure S43. IR-Spectrum of **3a**.

***6,7,8,9-tetrakis(3,3-dimethylbut-1-yn-1-yl)-4,11-bis((triisopropylsilyl)ethynyl)-5,10-dihydro-[1,2,5]thiadiazolo[3,4-b]phenazine* 3b-H_2**

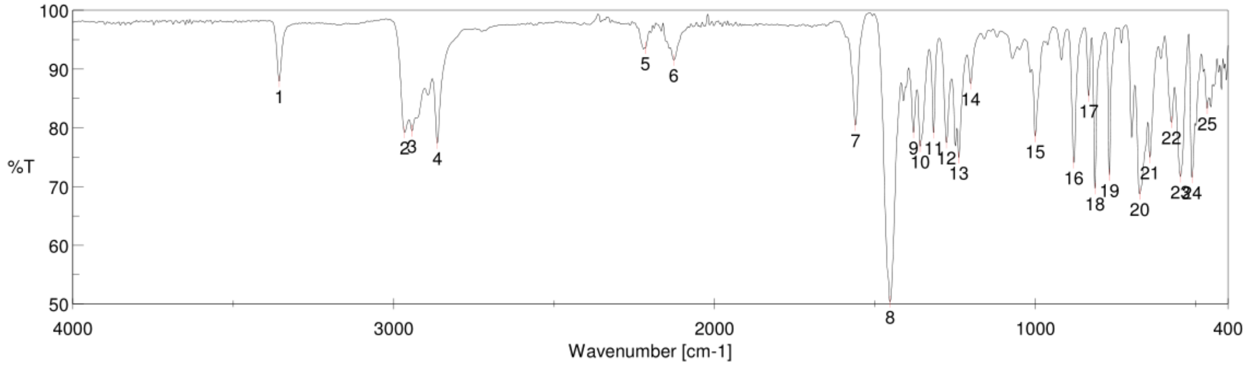

[Result of Peak Picking]

No.	Position	Intensity	No.	Position	Intensity	No.	Position	Intensity
1	3356.5	87.9146	2	2965.98	79.1848	3	2942.84	79.4707
4	2863.77	77.4013	5	2215.81	93.5449	6	2127.1	91.5429
7	1561.09	80.4396	8	1453.1	50.3572	9	1379.82	79.2035
10	1359.57	76.8456	11	1317.14	79.1501	12	1277.61	77.4571
13	1239.04	74.8933	14	1202.4	87.5048	15	1000.87	78.538
16	880.345	74.0589	17	834.062	85.4354	18	814.777	69.6437
19	769.458	71.9323	20	674.963	68.743	21	643.144	74.9531
22	576.612	80.9448	23	548.649	71.667	24	512.008	71.5343
25	464.761	83.2968						

Figure S44. IR-Spectrum of **3b-H_2**.

***6,7,8,9-tetrakis(3,3-dimethylbut-1-yn-1-yl)-4,11-bis((triisopropylsilyl)ethynyl)-[1,2,5]thiadiazolo[3,4-b]phenazine* 3b**

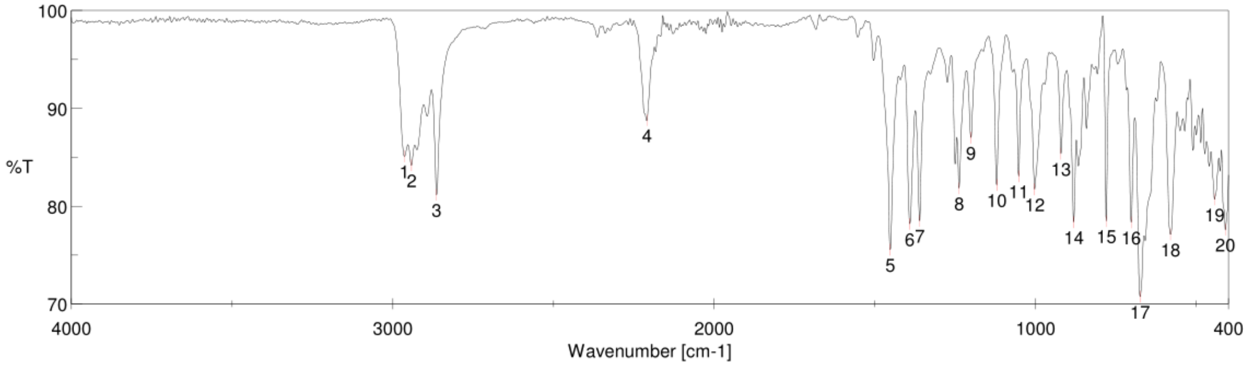

[Result of Peak Picking]

No.	Position	Intensity	No.	Position	Intensity	No.	Position	Intensity
1	2964.05	85.087	2	2941.88	84.1647	3	2863.77	81.1574
4	2209.06	88.7832	5	1452.14	75.5378	6	1391.39	78.1687
7	1360.53	78.4838	8	1238.08	81.8161	9	1201.43	86.9878
10	1121.4	82.152	11	1052.94	83.0264	12	1002.8	81.6937
13	920.843	85.3477	14	881.309	78.3559	15	780.065	78.4509
16	702.926	78.3363	17	674.963	70.728	18	580.469	77.1055
19	443.547	80.6735	20	409.799	77.5911			

Figure S45. IR-Spectrum of **3b**.

***1,4-bis((triisopropylsilyl)ethynyl)-6,7,8,9-tetrakis((trimethylsilyl)ethynyl)phenazine-2,3-diamine* 4a**

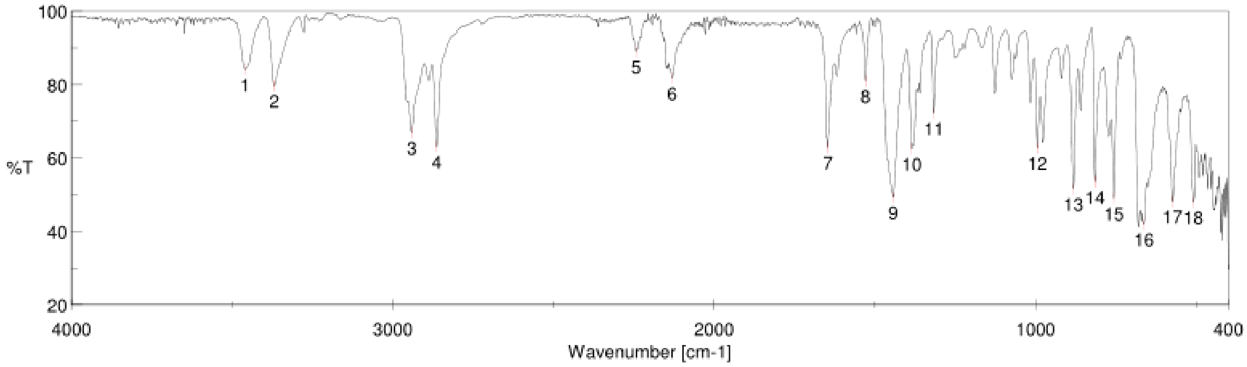

[Result of Peak Picking]

No.	Position	Intensity	No.	Position	Intensity	No.	Position	Intensity
1	3459.19	84.0572	2	3369.03	79.658	3	2940.91	66.9223
4	2863.29	63.0365	5	2242.81	88.9323	6	2130.47	81.6942
7	1646.43	62.7612	8	1529.27	80.9399	9	1442.98	49.279
10	1385.6	62.4678	11	1317.14	72.094	12	994.125	62.7044
13	882.756	51.4922	14	815.259	53.4662	15	756.441	48.9811
16	662.911	41.9234	17	573.719	48.1055	18	508.633	47.8854

Figure S46. IR-Spectrum of **4a**.

***6,7,8,9-tetrakis(3,3-dimethylbut-1-yn-1-yl)-1,4-bis((triisopropylsilyl)ethynyl)phenazine-2,3-diamine* 4b**

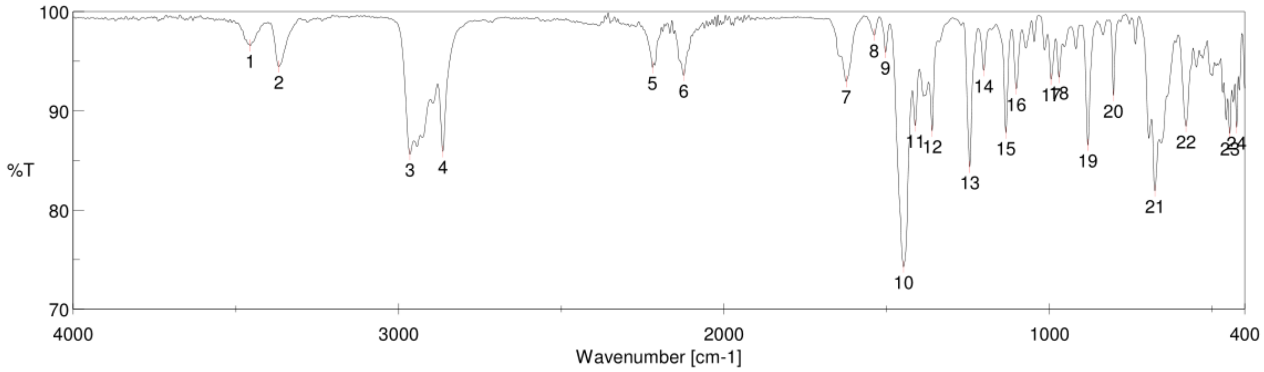

[Result of Peak Picking]

No.	Position	Intensity	No.	Position	Intensity	No.	Position	Intensity
1	3455.81	96.572	2	3368.07	94.4582	3	2965.02	85.6143
4	2863.77	85.8956	5	2219.67	94.3562	6	2124.21	93.5877
7	1624.73	92.9654	8	1538.92	97.6373	9	1504.2	95.908
10	1448.28	74.2731	11	1412.6	88.4936	12	1360.53	87.9697
13	1244.83	84.3172	14	1202.4	94.067	15	1133.94	87.7623
16	1102.12	92.2194	17	995.089	93.1905	18	970.983	93.3932
19	882.274	86.545	20	803.206	91.5446	21	675.928	81.9142
22	580.469	88.4399	23	445.476	87.7484	24	425.227	88.3114

Figure S47. IR-Spectrum of **4b**.

***2,3,5,12-tetrakis((triisopropylsilyl)ethynyl)-7,8,9,10-tetrakis((trimethylsilyl)ethynyl)-1,2-dihydropyrazino[2,3-b]phenazine* 6**

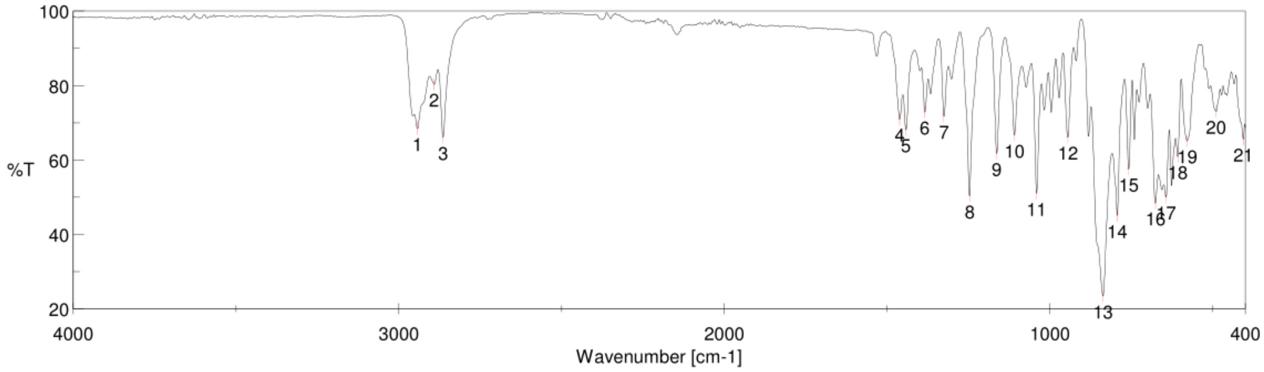

[Result of Peak Picking]

No.	Position	Intensity	No.	Position	Intensity	No.	Position	Intensity
1	2942.84	68.3954	2	2891.74	80.2756	3	2863.77	66.0058
4	1461.78	70.8935	5	1442.49	68.037	6	1384.64	72.8009
7	1325.82	71.6423	8	1246.75	50.2434	9	1163.83	61.7164
10	1108.87	66.5744	11	1041.37	50.8982	12	944.949	66.0964
13	836.955	23.3813	14	793.564	44.9833	15	757.888	57.4849
16	675.928	48.3399	17	643.144	49.9576	18	607.467	60.836
19	578.54	65.0471	20	489.831	73.0219	21	405.942	65.5448

Figure S48. IR-Spectrum of **6**.

***1,2,3,4-tetrabromo-6,13-bis((triisopropylsilyl)ethynyl)-8,9,10,11-tetrakis((trimethylsilyl)ethynyl)-quinoxalino[2,3-b]phenazine* 7a**

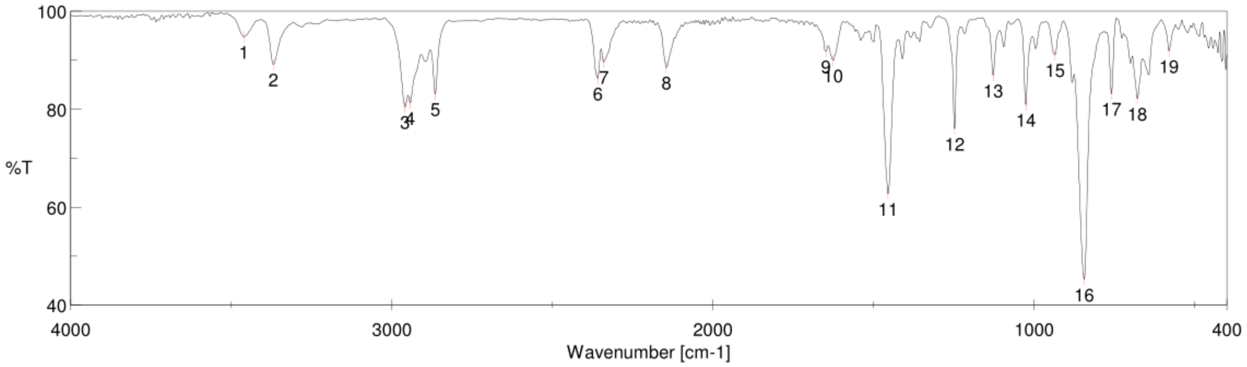

[Result of Peak Picking]

No.	Position	Intensity	No.	Position	Intensity	No.	Position	Intensity
1	3460.63	94.7488	2	3369.03	89.1031	3	2959.23	80.4445
4	2942.84	81.2734	5	2864.74	83.0575	6	2359.48	86.2804
7	2340.19	89.6264	8	2145.42	88.5059	9	1647.88	91.809
10	1625.7	89.9516	11	1455.03	62.6911	12	1247.72	75.96
13	1128.15	86.8895	14	1025.94	80.9233	15	935.306	91.052
16	844.669	45.2005	17	759.816	83.1102	18	678.82	82.1448
19	580.469	91.8873						

Figure S49. IR-Spectrum of **7a**.

***1,2,3,4-tetrabromo-8,9,10,11-tetrakis(3,3-dimethylbut-1-yn-1-yl)-6,13-bis((triisopropylsilyl)ethynyl)-5,14-dihydroquinoxalino-[2,3-b]phenazine* 7b-H$_2$**

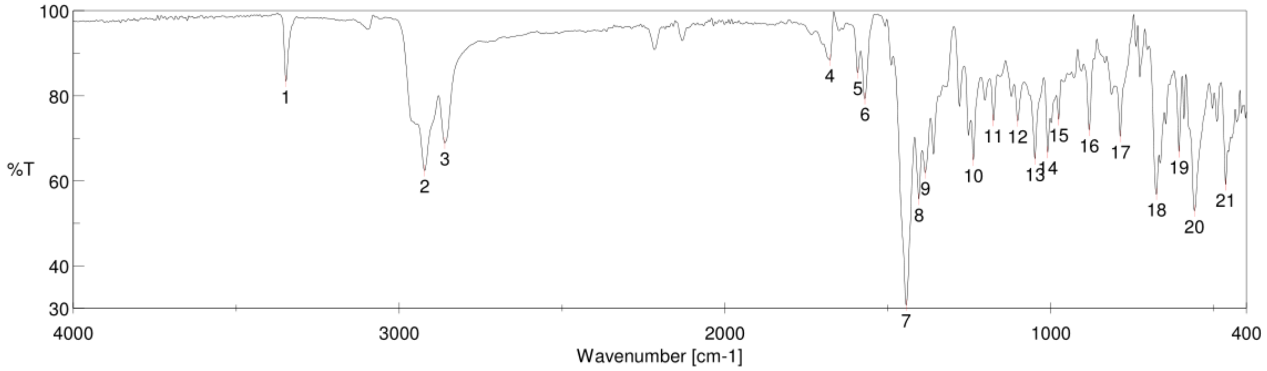

[Result of Peak Picking]

No.	Position	Intensity	No.	Position	Intensity	No.	Position	Intensity
1	3347.82	83.4104	2	2921.63	62.3839	3	2859.92	68.8847
4	1678.73	88.4099	5	1592.91	85.3973	6	1570.74	79.3305
7	1444.42	30.7093	8	1404.89	55.6316	9	1385.6	61.8858
10	1238.08	64.8514	11	1176.36	74.1998	12	1102.12	74.0721
13	1049.09	65.124	14	1009.55	66.689	15	975.804	74.4167
16	882.274	71.9082	17	786.815	70.4246	18	675.928	56.8389
19	606.503	66.8593	20	559.255	52.9244	21	463.796	59.082

Figure S50. IR-Spectrum of **7b-H$_2$**.

***1,2,3,4,8,9,10,11-octakis(3,3-dimethylbut-1-yn-1-yl)-6,13-bis((triisopropylsilyl)ethynyl)-5,14-dihydroquinoxalino[2,3-b]phenazine* 8-H₂**

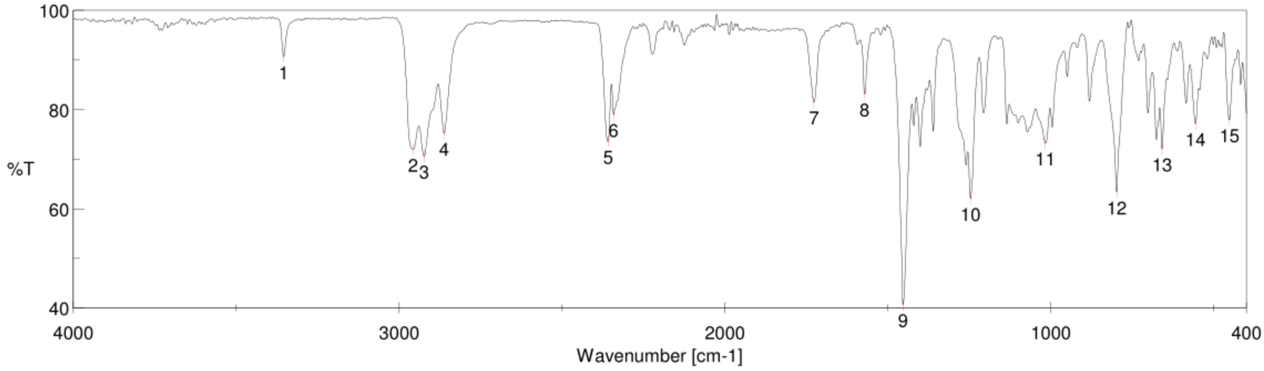

[Result of Peak Picking]

No.	Position	Intensity	No.	Position	Intensity	No.	Position	Intensity
1	3354.57	90.5914	2	2957.3	71.9378	3	2923.56	70.5474
4	2861.84	75.1866	5	2359.48	73.5297	6	2342.12	78.9488
7	1726.94	81.4599	8	1571.7	83.0736	9	1454.06	40.6346
10	1246.75	62.019	11	1017.27	73.1964	12	798.385	63.3202
13	658.571	72.0352	14	556.363	77.083	15	452.225	77.8672

Figure S51. IR-Spectrum of **8-H$_2$**.

***1,2,3,4,8,9,10,11-octakis(3,3-dimethylbut-1-yn-1-yl)-6,13-bis((triisopropylsilyl)ethynyl)-quinoxalino[2,3-b]phenazine* 8**

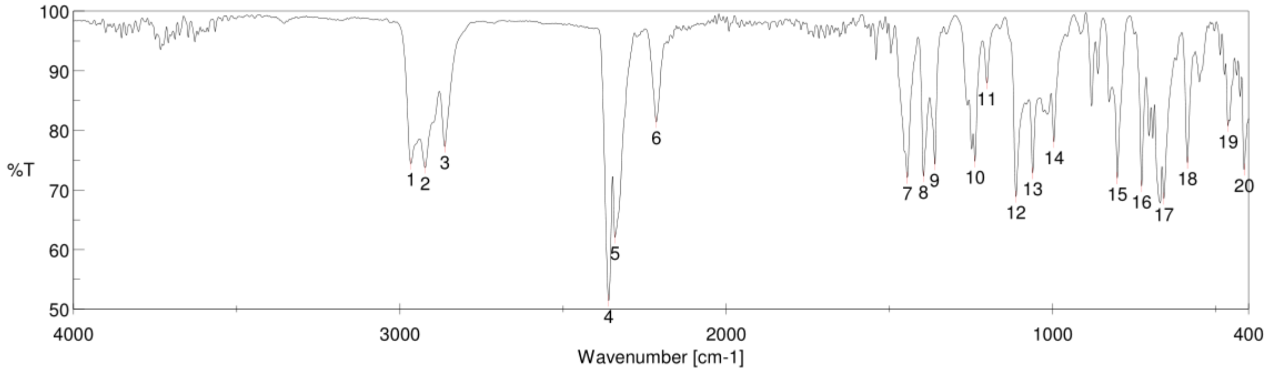

[Result of Peak Picking]

No.	Position	Intensity	No.	Position	Intensity	No.	Position	Intensity
1	2965.98	74.3961	2	2922.59	73.7373	3	2862.81	77.2553
4	2360.44	51.4052	5	2341.16	61.9754	6	2214.84	81.3689
7	1445.39	72.0328	8	1396.21	72.2811	9	1361.5	74.2614
10	1238.08	74.7664	11	1201.43	87.9008	12	1112.73	68.846
13	1061.62	72.7826	14	996.053	78.0651	15	802.242	71.9477
16	727.996	70.6019	17	659.536	68.4937	18	587.218	74.5409
19	462.832	80.703	20	413.656	73.3899			

Figure S52. IR-Spectrum of **8**.

Crystallographic Data

***2,3,5,12-tetrakis((triisopropylsilyl)ethynyl)-7,8,9,10-tetrakis((trimethylsilyl)ethynyl)-1,2-dihydropyrazino[2,3-b]phenazine* 6**

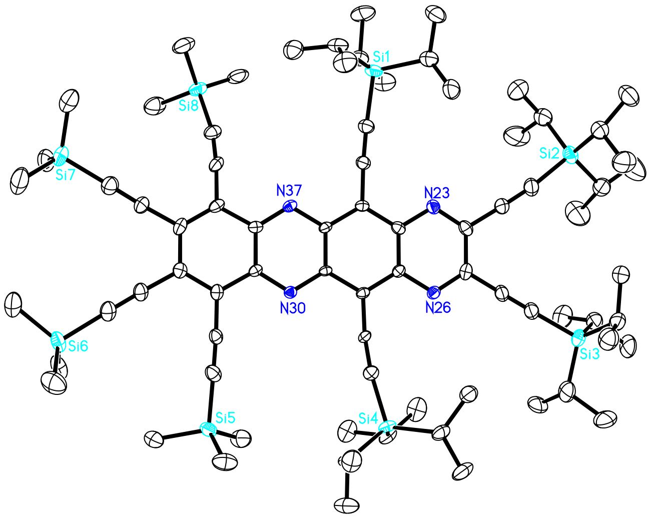

Identification code	hir26	
Empirical formula	$C_{79}H_{121}Cl_3N_4Si_8$	
Formula weight	1457.86	
Temperature	100(2) K	
Wavelength	1.54178 Å	
Crystal system	monoclinic	
Space group	$P2_1/c$	
Z	8	
Unit cell dimensions	a = 32.9037(5) Å	α = 90 deg.
	b = 14.3345(2) Å	β =101.969(1) deg.
	c = 38.9730(6) Å	γ = 90 deg.
Volume	17982.3(5) $Å^3$	
Density (calculated)	1.08 g/cm^3	
Absorption coefficient	2.24 mm^{-1}	
Crystal shape	plate	
Crystal size	0.129 x 0.107 x 0.071 mm^3	
Crystal colour	darkgreen	
Theta range for data collection	2.9 to 68.4 deg.	
Index ranges	$-39 \le h \le 37$, $-11 \le k \le 17$, $-46 \le l \le 42$	
Reflections collected	109356	
Independent reflections	31299 (R(int) = 0.0778)	
Observed reflections	17668 ($I > 2\sigma(I)$)	
Absorption correction	Semi-empirical from equivalents	
Max. and min. transmission	1.89 and 0.49	
Refinement method	Full-matrix least-squares on F^2	
Data/restraints/parameters	31299 / 393 / 1809	
Goodness-of-fit on F^2	0.92	
Final R indices (I>2sigma(I))	R1 = 0.067, wR2 = 0.162	
Largest diff. peak and hole	1.46 and -0.79 $eÅ^{-3}$	

Figure S53. Crystal data and structure of **6** (CCDC 1947891).

***1,2,3,4,8,9,10,11-octakis(3,3-dimethylbut-1-yn-1-yl)-6,13-bis((triisopropylsilyl)ethynyl)-quinoxalino[2,3-b]phenazine* 8**

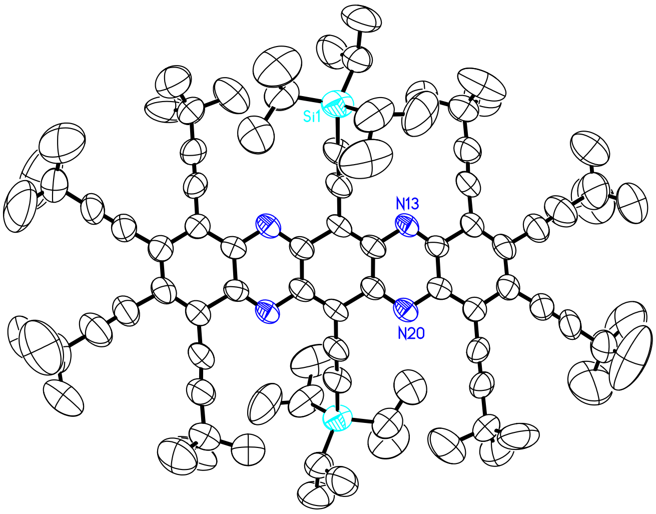

Identification code	hir30	
Empirical formula	$C_{88}H_{114}N_4Si_2$	
Formula weight	1284.01	
Temperature	200(2) K	
Wavelength	1.54178 Å	
Crystal system	monoclinic	
Space group	$P2_1/n$	
Z	2	
Unit cell dimensions	a = 20.607(4) Å	α = 90 deg.
	b = 11.2149(12) Å	β = 114.614(13) deg.
	c = 20.920(3) Å	γ = 90 deg.
Volume	4395.5(12) $Å^3$	
Density (calculated)	0.97 g/cm^3	
Absorption coefficient	0.67 mm^{-1}	
Crystal shape	plank	
Crystal size	0.222 x 0.043 x 0.021 mm^3	
Crystal colour	purple	
Theta range for data collection	2.5 to 47.8 deg.	
Index ranges	$-19\leq h\leq 19$, $-10\leq k\leq 9$, $-20\leq l\leq 19$	
Reflections collected	14830	
Independent reflections	4066 (R(int) = 0.1411)	
Observed reflections	2446 (I > 2σ(I))	
Absorption correction	Semi-empirical from equivalents	
Max. and min. transmission	2.05 and 0.53	
Refinement method	Full-matrix least-squares on F^2	
Data/restraints/parameters	4066 / 624 / 425	
Goodness-of-fit on F^2	2.31	
Final R indices (I>2sigma(I))	R1 = 0.170, wR2 = 0.368	
Largest diff. peak and hole	0.35 and -0.37 $eÅ^{-3}$	

Figure S54. Crystal data and structure of **8** (CCDC 1947892).

References

[1] G. R. Fulmer, A. J. M. Miller, N. H. Sherden, H. E. Gottlieb, A. Nudelman, B. M. Stoltz, J. E. Bercaw, K. I. Goldberg, *Organometallics* **2010**, *29*, 2176-2179.

[2] Gaussian 09, Revision D.01, M. J. Frisch, G. W. Trucks, H. B. Schlegel, G. E.Scuseria, M. A. Robb, J. R. Cheeseman, G. Scalmani, V. Barone, B. Mennucci, G. A. Petersson, H. Nakatsuji, M. Caricato, X. Li, H. P. Hratchian, A. F. Izmaylov, J. Bloino, G. Zheng, J. L. Sonnenberg, M. Hada, M. Ehara, K. Toyota, R. Fukuda, J. Hasegawa, M. Ishida, T. Nakajima, Y. Honda, O. Kitao, H. Nakai, T. Vreven, J. A. Montgomery, Jr.,J. E. Peralta, F. Ogliaro, M. Bearpark, J. J. Heyd, E. Brothers, K. N. Kudin, V. N. Staroverov, R. Kobayashi, J. Normand, K. Raghavachari, A. Rendell, J. C. Burant, S. S. Iyengar, J. Tomasi, M. Cossi, N. Rega, J. M. Millam, M. Klene, J. E. Knox, J. B. Cross, V. Bakken, C. Adamo, J. Jaramillo, R. Gomperts, R. E. Stratmann, O. Yazyev, A. J. Austin, R. Cammi, C. Pomelli, J. W. Ochterski, R. L. Martin, K. Morokuma, V. G. Zakrzewski, G. A. Voth, P. Salvador, J. J. Dannenberg, S. Dapprich, A. D. Daniels, Ö. Farkas, J. B. Foresman, J. V. Ortiz, J. Cioslowski, D. J. Fox, Gaussian, Inc., Wallingford CT, **2009**.

[3] Y.-T. Wu, D. Bandera, R. Maag, A. Linden, K. K. Baldridge, J. S. Siegel, *J. Am. Chem. Soc.* **2008**, *130*, 10729-10739.

Author Contributions

((Please specify the contributions of each author including the type (e.g. data curation, funding acquisition, formal analysis, investigation, project administration, validation, writing of original draft) and the degree (e.g. lead, equal, supporting) of contribution.))
